# Quantum Interference and Contact Effects in the Thermoelectric Performance of Anthracene-Based Molecules

**DOI:** 10.1021/acs.jpcc.3c00069

**Published:** 2023-04-10

**Authors:** Joseph M. Hamill, Ali Ismael, Alaa Al-Jobory, Troy L. R. Bennett, Maryam Alshahrani, Xintai Wang, Maxwell Akers-Douglas, Luke A. Wilkinson, Benjamin J. Robinson, Nicholas J. Long, Colin Lambert, Tim Albrecht

**Affiliations:** † School of Chemistry, University of Birmingham, Edgbaston Campus, Birmingham B15 2TT, U.K.; ‡ Physics Department, Lancaster University, Lancaster LA1 4YB, U.K.; § Department of Physics, College of Science, University of Anbar, Ramadi 31001, Anbar, Iraq; ∥ Department of Chemistry, Imperial College London, MSRH, White City, London W12 0BZ, U.K.; ⊥ Physics Department, College of Science, University of Bisha, P.O. Box 344, Bisha 61922, Kingdom of Saudi Arabia; # School of Information Science and Technology, Dalian Maritime University, Dalian 116026, China

## Abstract

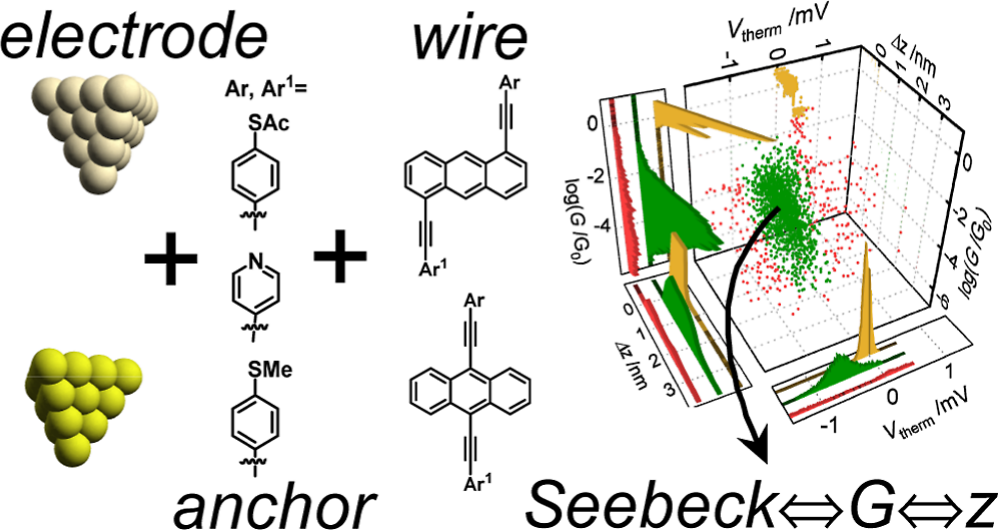

We report on the single-molecule electronic and thermoelectric properties of strategically chosen anthracene-based molecules with anchor groups capable of binding to noble metal substrates, such as gold and platinum. Specifically, we study the effect of different anchor groups, as well as quantum interference, on the electric conductance and the thermopower of gold/single-molecule/gold junctions and generally find good agreement between theory and experiments. All molecular junctions display transport characteristics consistent with coherent transport and a Fermi alignment approximately in the middle of the highest occupied molecular orbital/lowest unoccupied molecular orbital gap. Single-molecule results are in agreement with previously reported thin-film data, further supporting the notion that molecular design considerations may be translated from the single- to many-molecule devices. For combinations of anchor groups where one binds significantly more strongly to the electrodes than the other, the stronger anchor group appears to dominate the thermoelectric behavior of the molecular junction. For other combinations, the choice of electrode material can determine the sign and magnitude of the thermopower. This finding has important implications for the design of thermoelectric generator devices, where both n- and p-type conductors are required for thermoelectric current generation.

## Introduction

**Figure 1:**
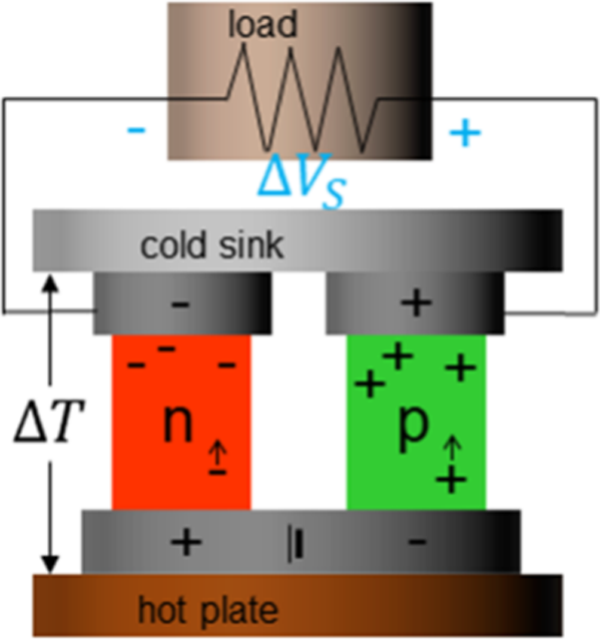
Schematic of a thermoelectric generator with the n- and p-conducting branches, cold and hot reservoirs, load resistance, and the thermal (Seebeck) voltage.

Thermoelectric power generation has interesting prospects because it is one of only a few methods to convert waste heat into electrical energy in a low-maintenance, robust device format. The basic setup of a thermoelectric generator is shown in Figure 1, including the p- and n-type semiconducting branches, the temperature gradient, the resulting Seebeck voltage Δ*V*_s_, as well as the load resistance.^1,2^ However, one of the disadvantages of the technology is that its efficiency is relatively low, fundamentally due to the Carnot limit but also because of the limitations imposed by the materials used.^3,4^ To this end, the material-specific figure of merit ZT may be defined as shown in eq 1
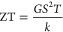
1where *G* is the electrical conductance, *S* is the thermopower, and *k* = *k*_e_ + *k*_p_ is the thermal conductance, which is the sum of the contributions from electrons (*k*_e_) and phonons (*k*_p_). Maximizing ZT requires the simultaneous maximization of *S* and *G*/*k* = *GT*/*k*_e_(1 + *k*_p_/*k*_e_). Since the Wiedemann–Franz law states that *GT*/*k*_e_ = 1/*L*, where *L* is the Lorentz number, which is independent of materials parameters and temperature, maximizing ZT requires *k*_p_/*k*_e_ ≪ 1. Indeed, achieving ZT > 1 at room temperature has proven to be challenging, and only in recent years, materials with larger ZT values have been found.^5−7^ These recent breakthroughs are typically achieved through careful nanostructuring of known materials,^8−10^ but there is a strong need for the discovery of new ones with significantly better performance. Indeed, organic materials have been identified as promising candidates, with evidence suggesting that *G*, *S,* and *k* may be optimized independently to some degree, at least in some charge transport regimes.^11−18^

An additional aspect is that many thermoelectric materials, old and new, contain elements on the EU’s list of critical at-risk raw materials—*e.g.,* bismuth, hafnium, and antimony.^19^ This list was compiled as part of work by the EU to identify those elements that were at risk of becoming scarce as a result of, *e.g.,* supply chain breakdown. In the cases of bismuth and antimony, this risk is in part a consequence of the fact that up to 80% of the world’s supply comes from only one country, and, as with bismuth, it is rarely recycled or recyclable. Telluride is a common component of many of these materials, and although it is not on this list, it is as rare as platinum in the Earth’s crust.^5^ Organic molecules, on the other hand, may be synthesized using sustainable feed stock, which is not threatened by supply chain stresses, and with greater design flexibility beyond transition metal crystalline geometries. However, for organic thermoelectric power generation to become viable, it has been argued that eight milestones must be met.^20,21^ The first four of which are to achieve:

(1) a power factor *GS*^2^ > 10^4^ aW K^2^
(2) a phonon thermal conductance *k*_p_ < 10 pW K^–1^
(3) reproducible predictions and measurements of Seebeck coefficients and electrical and thermal conductances for systems with thermoelectric figures of merit ZT > 3
(4) achieve comparable single-molecule and small-area predictions and measurements

**Figure 2:**
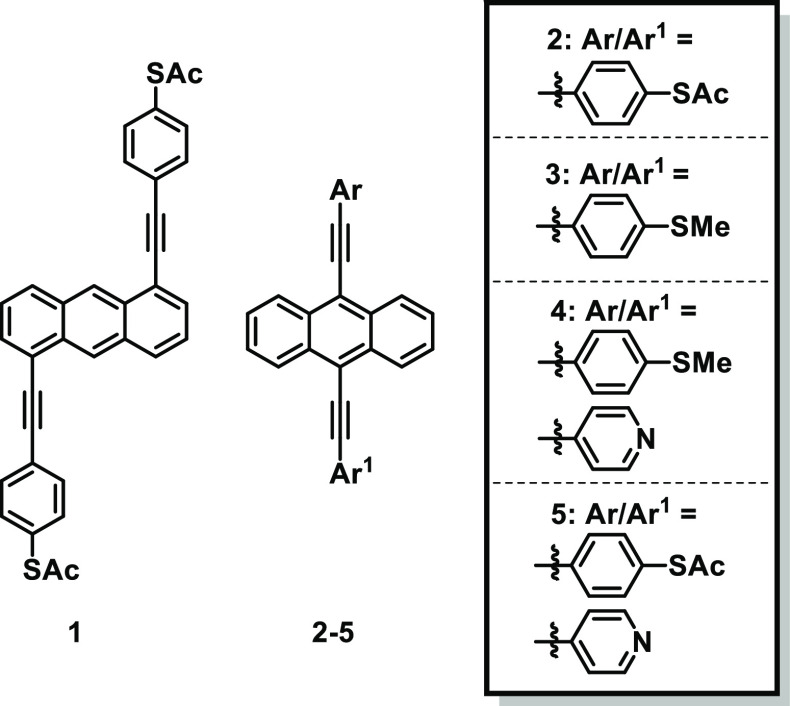
(a) Molecules measured in this study: 1,5-di(4-(ethynylphenyl)thioacetate)anthracene (**1**), 9,10-di(4-(ethynylphenyl)thioacetate)anthracene (**2**), 9,10-di(4-ethynylthioanisole)anthracene (**3**), 9-(4-(ethynylphenyl)thioacetate)-10-(4-ethynylpyridine)anthracene (**4**), and 9-(4-ethynlthioanisole)-10-(4-ethynylpyridine)anthracene (**5**).

The remaining milestones are concerned with scaling-up the achievements of the first four milestones. Our present study mainly relates to milestones 1, 3, and 4 based on a quick and effective method for characterizing single-molecule electric conductance and Seebeck coefficients.^22^ Here, we set out to utilize this method to explore their dependence on molecular connectivity and anchor groups for a set of anthracene-based molecules, Figure 2, which are known to feature quantum interference effects.^23,24^ Supported by theoretical calculations and comparison to previously reported thin-film results, we explore the effect of the nature of the anchor groups in combination with the substrate material. Interestingly, apart from variations in the magnitude of the Seebeck coefficient, we have observed a sign reversal resulting from a change in junction from Au/Pt to Au/Au. While such behavior has been observed before for benzenedithiol in Au/Au and Au/Ni junctions and has been rationalized based on spin hybridization at the Fermi level,^25^ in our case, the change in sign appears to reflect a more subtle difference in the bonding interaction between the anchor groups and the substrate electrodes, with concomitant changes in Fermi level alignment.

## Methods

Chemicals and syntheses of molecules **1–5** are shown in Figure 2. We have previously reported the synthesis of compounds **1–4** and refer the reader to ref (23) for further details of their synthesis and characterization. Compound **5** was synthesized by employing a stepwise Sonogashira methodology utilizing reactions between 9,10-dibromoanthracene and terminal alkynes. 4-(Ethynylphenyl)thioacetate can undergo self-oligomerization to form a cyclic trimer when exposed to Sonogashira conditions.^26^ In order to avoid this unwanted side reaction, we decided to utilize a protecting-group strategy. Our previous work utilized a *tert*-butyl protecting group which could be interconverted to a thioacetate through treatment with boron tribromide to allow for dealkylation, followed by quenching with acetic anhydride. In our experience, however, attempts to apply this methodology in the synthesis of compound **5** were unsuccessful. Considering this, we moved to the use of a cyanoethyl-protected thiol, which presents much milder deprotection conditions. To this end, we synthesized 4-(ethynylphenyl)-thiocyanoethyl following the methodology presented by Bryce *et al.* and subsequently reacted this with 9,10-dibromoanthracene in a 1:5 ratio under Sonogashira conditions.^27^ This reaction generated a mixture of the monosubstituted (**5A**, see Supporting Information S1.2) and symmetrically disubstituted products (**5B**), which could be trivially separated from one another using flash chromatography. The monosubstituted product was subsequently reacted with 4-ethynylpyridine under analogous conditions to produce an asymmetrically disubstituted product (**5C**). The final step involved interconversion of the thiol-protecting group through first treating compound (**5C**) with sodium methoxide to allow for removal of the cyanoethyl group before quenching with acetic anhydride to generate a terminal thioacetate. This was purified using an aqueous work-up to provide compound (**5**) in good yield. Further details can be found in Section S1 of the Supporting Information.

**Figure 3:**
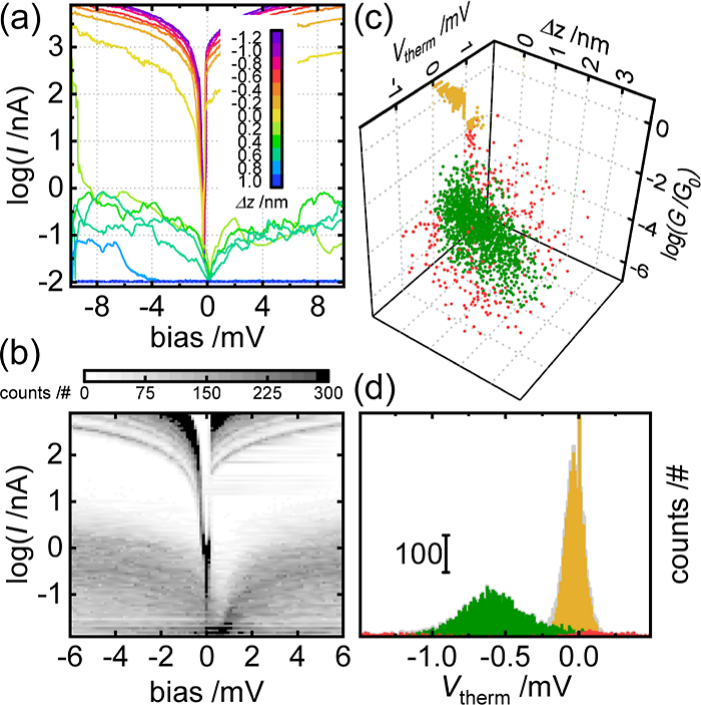
Example STM IV experiment performed on the adlayer of molecule **2**. (a) Example withdrawal series with *I*/*V* sweeps across the Au/Au junction in purple through yellow. Sweeps across Au/**2**/Au in green. Sweeps across the open junction in blue. (b) 2D current *vs* bias intensity plot after *V*_corr_ is removed from each group. (c) 3D scatter plot of displacement, Δ*z*, conductance, *G*, and voltage offset, *V*_therm_, from each trace (sweeps across the open junction are removed) clustered using the Gaussian mixture model into a Au/Au cluster (gold) and a molecular cluster (green) and a noise cluster (red). (d) 1D histograms of *V*_therm_ for the three clusters above and for the entire data set (gray) at Δ*T* = 27 K.

For the determination of single-molecule Seebeck coefficients, a distance-dependent scanning tunneling microscopy (STM) current–voltage (*I*/*V*) method was used.^22^ Briefly, the tip was first brought into contact with the substrate surface and then withdrawn in 25 steps of 0.2 nm (in some experiments 0.3 nm). During each step, the bias voltage was swept between ±10 mV at a rate of 0.2 V s^–1^ and the current was recorded (tip withdrawal rate: ∼2 nm s^–1^, 2.5 s per series), *cf.*Figure 3a as an example. Typically, three different classes of *I*/*V* sweeps were observed, namely Au/Au (purple to yellow), Au/molecule/Au (green), and open junctions (blue). The conductance *G* for each *I*/*V* sweep was determined based on 41 data points centered at −5 mV. The sweep with a conductance closest to but larger than the quantum conductance *G*_0_ was taken to define the voltage correction *V*_corr_ for each *I*/*V* sweep within a given series. At each Δ*T*, *ca.* 1000 withdrawals and thus 25 000 *I*/*V* traces were recorded. The sweeps were parameterized into the three-dimensional space (Δ*z*, *G*, Δ*V*), Figure 3c, and clustered into three clusters using a Gaussian mixture model.^28,29^ To illustrate the voltage shift due to Δ*T*, the 1D histogram of Δ*V* values for molecule **2** at Δ*T* = 27 K is shown in Figure 3d: sweeps assigned to Au/Au junctions (yellow) are tightly centered around 0 μV, sweeps assigned to noise (red) widely distributed while sweeps assigned to Au/molecule/Au sweeps (green) show a clear offset of 0.5 mV.

**Figure 4:**
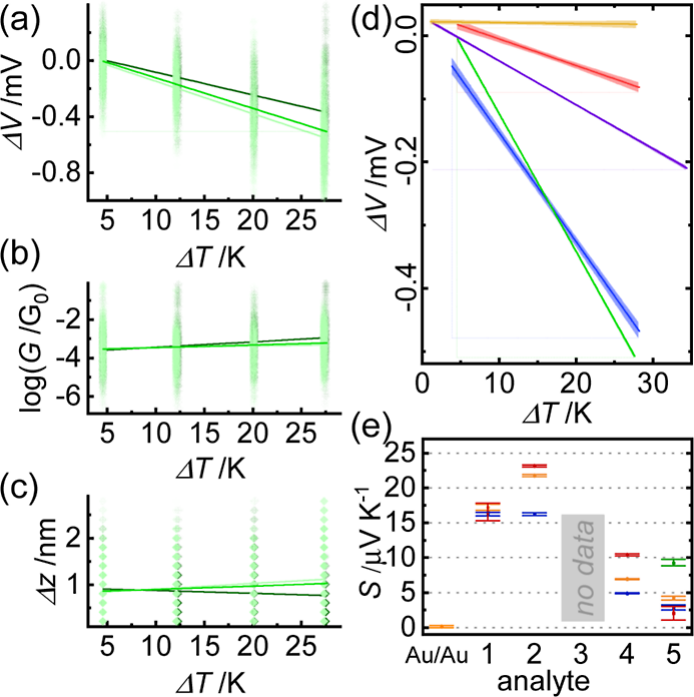
(a) Scatter plots of Δ*V vs* Δ*T* measurements of molecule **2** with separate trend lines for two separate experiments (light and dark green) and combined trend lines with 95% confidence intervals (green). (b) Scatter plots of *G vs* Δ*T* and (c) Δ*z vs* Δ*T* for the same measurements, with *G*_mol_ and Δ*z*_mol_ from each separate measurement calculated as the mean of a Gaussian fit of all data and standard deviation as error bars. Trend lines are aids for the eye. (d) Δ*V vs* Δ*T* trend lines with 95% confidence intervals for molecules **1** (blue), **2** (green), **4** (purple), **5** (red), and clean Au/Au (gold). (e) Summary of *S*_mol_ for all molecules in this study, and the internal reference at the Au/Au contact. Blue/red/green represent trials 1/2/3, and orange is the combined result (error bars: standard error of the slope from the linear least-square fit).

**Figure 5:**
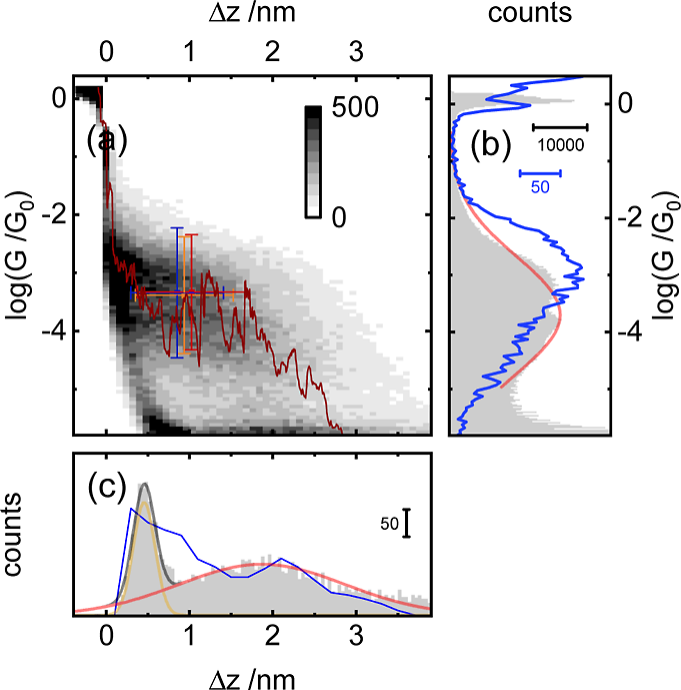
Example constant bias STM BJ experiment performed on the adlayer of molecule **2**. (a) *ca.* 7k traces combined in a 2D conductance *vs* displacement intensity plot with an example trace (crimson). Cross-hairs are (Δ*z*_mol_, *G*_mol_) from the STM IV measurements with a standard deviation [trials 1 and 2 (blue), combined data set (orange)]. (b) 1D conductance histogram of all traces from constant bias measurements in gray and the Gaussian fit of the molecular plateau region (red). Blue: 1D conductance histogram of all sweeps from the STM IV mode from data plotted in Figure 3c. (c) 1D displacement histogram of all traces in constant bias measurement determined at a conductance of 10^–5.2^*G*_0_. Two-peak area-type Gaussian fit (red/yellow). Junction formation probability: 77%. 1D displacement histogram from the molecular cluster in STM IV mode (blue line) from data plotted in Figure 3c.

For each molecule, experiments were conducted at 4–10 different Δ*T* values, and results for each analyte were replicated on different days. Each of the three parameters was plotted *vs* Δ*T*, Figure 4, and each replicate was fitted separately (light and dark red lines in Figure 4a–c). A combined linear fit was calculated to determine the overall slope and the standard error of the slope and plotted along with a 95% confidence interval, Figure 4d, for the voltage correction (at Au/Au contact)^22^ as well as molecules **1**, **2**, **4,** and **5**. Figure 4e shows *S*_mol_ for each replicate (blue/red/green) and an overall *S*_mol_ (orange) (error bars: standard error of slope), *cf.* also Figures S9 and S10 in the Supporting Information. Constant bias STM BJ measurements were also performed on all analytes to compare with results from STM IV measurements and to potentially gain additional insight into the junction geometry and progression.^22^ In brief, the STM tip is initially brought into contact with the substrate surface at a constant tip/substrate bias (here: 100 mV). It is then withdrawn at a constant rate, typically between 8 and 16 nm s^–1^, and the current is recorded. A typical withdrawal trace is plotted in Figure 5a in crimson for a measurement of molecule **2**, showing the range from the Au/Au contact to the noise level. The region where charge transport through the molecular bridge dominates is indicated by a plateau-like region. The intensity plot of *ca.* 7400 traces in Figure 5a exhibits both tunneling traces (“empty” gaps) and molecular traces. The tunneling traces are evident by the dense cloud of short traces, which decay linearly between 10^–4^ and 10^–5^*G*_0_. The molecular traces show more variation and exhibit a broadly distributed plateau region at 10^–3.5^*G*_0_ that extends for about 2 nm. The mean conductance *G*_mol_ of the molecular plateau was determined from a Gaussian fit (red) of the 1D histogram of conductance values, Figure 5b. The tunneling traces contributed negligibly to the conductance histogram because they exhibit few data points in the molecular region. To determine the plateau length, the distance between *G* = *G*_0_ and *G* ≤ 10^–5.2^*G*_0_, was determined for each trace. A histogram of the plateau lengths, in gray in Figure 5c, yielded two peaks. The first peak at *ca.* 0.5 nm was due to rapidly decaying tunneling traces (yellow), while the second peak (red), at *ca.* 2.0 nm, is related to molecular junctions [77%, based on the relative area of the Gaussian fits. For comparison, Δ*z*_mol_ and *G*_mol_ values from STM IV measurements are depicted (replicates in red/blue, combined data set in orange; error bars: standard errors of the means). Hence, the close mapping of the STM IV results onto the STM BJ results was a strong confirmation that the molecules were present in the STM IV measurements and that the clustering step of the analysis was selecting for molecular *I*/*V* sweeps. See Figures S11–S13 in the Supporting Information for STM BJ results for molecules **1**, **4**, and **5**].

The transport properties of the studied junctions were further investigated using a combination of density functional theory (DFT) and quantum transport theory^30^ to obtain the transmission coefficient *T*(*E*) describing electrons of energy *E* passing from the source to the drain electrodes.^31^ Using the density functional code SIESTA, the optimum geometries of isolated molecules were obtained by relaxing the molecules until all forces on the atoms were less than 0.01 eV/Å.^32,33^ A double-ζ plus polarization orbital basis set, norm-conserving pseudopotentials, and an energy cut-off of 250 rydbergs defining the real space grid were used, and the local density approximation (LDA) was chosen as the exchange correlation functional. We also computed results using GGA and found that the resulting transmission functions were comparable with those obtained using LDA.^34−36^ To calculate the optimum binding distance between a molecule and an electrode, we used DFT and the counterpoise method, which removes basis set superposition errors. The binding distance *d* is defined as the distance between molecule A and electrode B. The ground state energy of the total system is calculated using SIESTA and is denoted as *E*_AB_^AB^. The energy of each entity is then calculated on a fixed basis, which is achieved using ghost atoms in SIESTA. Hence, the energy of A in the presence of the fixed basis is defined as *E*_A_^AB^ and for the electrode B as *E*_B_^AB^. The binding energy is then calculated using the following equation: BE = *E*_AB_^AB^ – *E*_A_^AB^ – *E*_B_^AB^. Transmission coefficient curves *T*(*E*) were obtained using the GOLLUM transport code.^30^ Following this, the Seebeck coefficient (*S*) of the junction was calculated as described in Section S3 of the Supporting Information.

## Results and Discussion

The results for molecules **1–5** are summarized in Table 1, for both STM IV and STM BJ methods (top and bottom values in each row), and column-wise from left to right, Δ*z*_mol_, *G*_mol_, *S*_mol,_ and the power factor *f*. We note that for **3**, we were unable to obtain reproducible results using the STM IV method, and hence, no thermopower value could be determined. The final row represents nominal results from “empty” tunneling junctions, *i.e.,* in the absence of a molecular bridge, as described in further detail in ref (22). The data lend themselves to several broad observations: (1) Δ*z*_*mol*_ values are usually found to be close to or just below 1 nm, which is shorter than the value of approximately 2 nm expected for fully extended bridges of these molecules. Exceptions are the values determined for **1** and **2** using STM BJ, where the Δ*z*_mol_ values are in good agreement with theoretical expectations. Both molecules feature thiol-based anchor groups, which form strong bonds to the respective gold electrode contacts. Hence, the observed difference in Δ*z*_mol_ between the two methods has been somewhat unexpected. It is unlikely due to a difference in anchor points, given the strong affinity between the thiol groups and the gold surfaces and the absence of other competitive binding sites in the molecules. It can also not be rationalized solely on the basis of the smaller spatial resolution in the STM IV measurement (step size between *I*/*V* sweeps: 0.2–0.3 nm) or junction rupture based on differences in the applied bias voltage (which is smaller for STM IV). One notable difference between the two methods, as implemented here, is, however, in the time required to record a current–distance characteristic, given the withdrawal rates in STM BJ (8–16 nm s^–1^) and STM IV (2 nm s^–1^). Accordingly, the time required to fully extend the molecular junction to 2 nm is between 0.125 and 0.25 s (for STM BJ) and about 1 s (for STM IV). It is then conceivable that in the presence of a thermal or mechanical drift, the molecular junction ruptures prematurely in relatively slow STM IV measurements, while the full molecular extension is reached during faster STM BJ recordings.

**Table 1:** Results from Current–Distance Spectroscopy at Constant Bias (Top) and Distance-Dependent *I*/*V* Spectroscopy (Bottom)^a^

molecule	Δ*z*_mol_ (nm)	*G*_mol_ [log(*GG*_0_^–1^)]	*S*_mol_ (μV K^–1^)	*f* (aW K^–2^)
**1** (1,5 SAc2)	2(1)	–5(1)		
	0.8(5)	–4(1)	17.2(4)	4.5
**2** (9,10 SAc2)	2(2)	–4(2)		
	0.9(6)	–3(1)	21.8(1)	16
**3** (9,10 SMe2)	1(1)	–3(2)		
**4** (9,10 SMe,N)	1.1(3)	–4.0(9)		
	0.8(4)	–4(1)	6.95(7)	0.70
**5** (9,10 SAc,N)	1(1)	–4(1)		
	0.7(5)	–4(1)	4.2(3)	0.41
**Au/Au**	0.4(2)	–3(1)	[−0.2(2)]	

a Values for Δ*z*_mol_ and *G*_mol_ are the sample mean and standard deviation for all Δ*T* and all replicates; *S* values are the slope and standard error of the slope for the trend line through all Δ*T* and all replicates.

Similar considerations may apply to the remaining molecules **3–5**, but now both methods yielded shorter than expected Δ*z*_mol_ values of about 1 nm (STM BJ, **3–5**) and around 0.8 nm (STM IV, **4**/**5**). These molecules all contain at least one SMe or pyridyl anchor group, and our previous X-ray photoelectron spectroscopy studies indeed suggest that their interaction with the Au surface is comparable but weaker than the thiol/Au interaction.^37^ It is therefore possible that the apparent break-off distance is affected by the lifetime of the molecular bridge, which does not fully extend before being ruptured. In this context, we also considered whether an alternative contact geometry, for example, *via* the anthracene unit, could explain our observations. While at first glance, consistent with shorter Δ*z*_mol_ values, it would imply that the well-defined anchor groups are not involved in bridging the electrode gap, despite their surface geometry and known affinity to gold, in contradiction to our modeling results. At the same time, while the anthracene moiety may interact with gold directly, the interaction strength is relatively low,^38^ making it unlikely that it dominates junction formation at the expense of the well-known anchor groups used in this study. Overall, this scenario therefore appears less likely, even though further systematic studies may be required to explore the effect of junction stability and dynamics.

(2) With regards to *G*_mol_, for **1** and **2** STM BJ yielded smaller values than STM IV, and in conjunction with the longer break-off distance, this might suggest a non-negligible contribution from other conductance pathways, namely “through-space” tunneling^39^ Comparing *G*_mol_ for **1** and **2** for the same spectroscopic method, we find the value for **2** to be about 1 order of magnitude larger than for **1**, broadly in line with expectations from magic ratio theory as a result of quantum interference effects, see also Figures S22 and S23 in the Supporting Information.^23,40^ Despite our best efforts, we have been unable to obtain a *G*_mol_ value for **3** using STM IV, but for **4** and **5**, both spectroscopic methods yielded the same values within the experimental error. None of the molecules appears to be particularly conductive, although with *G*_mol_ values smaller than −3 in logarithmic units of *G*_0_. (3) The *S*_mol_ values were determined successfully for **1**, **2**, **4,** and **5**, where those for **1** and **2** are similar and significantly higher than those for **4** and **5**. This could suggest that in the latter two cases, the Fermi level is closer to the center of the highest occupied molecular orbital/lowest unoccupied molecular orbital (HOMO/LUMO) gap. The magnitudes *G*_mol_ and *S*_mol_, and hence the power factor *f*, are, however, small for all molecules studied here in comparison to the milestones listed above. Even for the best performing molecule **2**, the value of *f* = 16 is still significantly below the stipulated value of 10^4^. Further optimization of both *G*_mol_ and *S*_mol_ is therefore required, for example, by the careful design of the electronic structure of the junction or electrostatic gating.^41^

**Figure 6:**
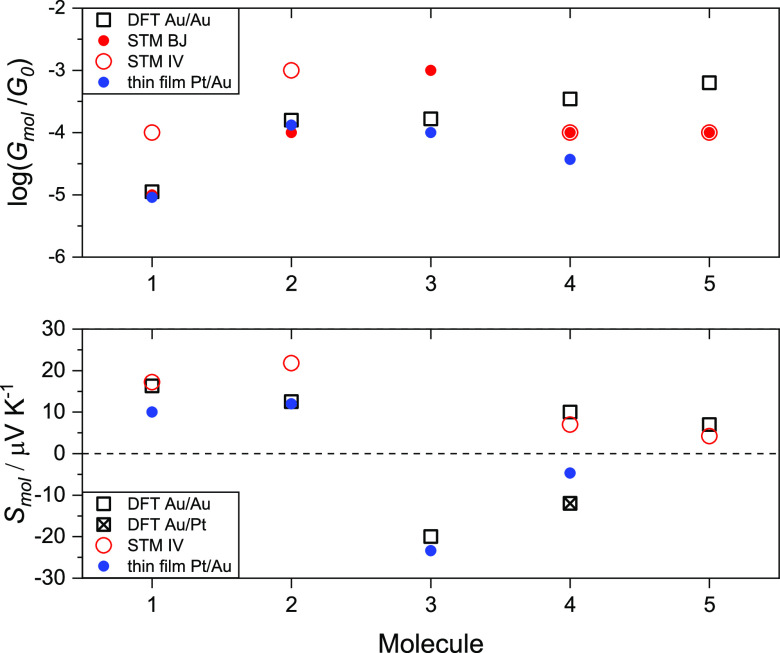
Electric and thermoelectric properties of **1–5**, a comparison between the experiment and theory. Top panel: *G*_mol_ values from theory in Au/Au and Au/Pt junctions for **1–5** are similar at DFT-predicted mid-gap (black squares). Experiments for **1** and **2** formally yield a high *G*_mol_ value (STM IV, red open circles) and a lower one (solid red circles), see above, which is closer to theoretical predictions. Experimental *G*_mol_ values for **1–4** extracted from thin film measurements in Au/Pt junctions (blue circles) from refs (23) and (24) are shown for comparison and compare well with theoretical predictions and values from STM BJ. Bottom panel: predicted *S*_theory_ in Au/Au junctions (black squares) for **1**, **2**, **4,** and **5** is positive, whereas for **3,** it is negative, in line with both experimental data sets, where available. Interestingly, for **4**, STM IV data from Au/Au junctions yield a positive *S*_mol_ value but a negative one for measurements in Pt/Au junctions. This can be rationalized based on substrate-induced changes in the electronic structure of the junction, as described in the main text. Note: Experimental *S*_mol_ values are unavailable for **3** and **5** in Au/Au and Au/Pt junctions, respectively; theoretical values are all mid-gap simulations (*E*_F_ – *E*_F_^DFT^ ≈ mid-gap).

Finally, all molecules showed positive *S*_mol_ values, suggesting that charge transport is HOMO-dominated and likely due to the sulfur-based anchor groups, further supporting the interpretation of the Δ*z*_mol_ data presented above. Barring one exception, the magnitude of *S*_mol_ is comparable to previously reported values for molecules **1**, **2,** and **5** determined in Au/Pt thin-film junctions, see refs (23) and (24) and Figure 6. The exception is **4**, where we find *S*_mol_ > 0, while previous work in Au/Pt thin-film devices yielded *S*_mol_ < 0. This would imply a change in the charge transport mechanism from hole-dominated to electron-dominated transport and may be induced by a slight shift of the Fermi level offset, *e.g.,* due to differences in the interaction between the anchor groups and the respective substrate materials (Au/Au *vs* Au/Pt).

To explore the electronic structure of the junctions and the effects of the substrate metal and anchor groups on *S*_mol_ in more detail, we undertook a detailed DFT study, see Section S3 in the Supporting Information for details, which led to the following main conclusions. First, the simulations show that thiol-terminated anthracene binds about 2 times stronger to an Au electrode than a pyridyl/SMe-terminated anthracene (with binding energies approximately 1.0 *vs* 0.5 eV), *cf.*Figures S16 and S17–S22 for the optimized structures of the respective junctions. Second, we found good agreement between theory and experiment, in terms of *G*_mol_ and the sign and magnitude of *S*_mol_, if the Fermi energy is taken to be near the middle of the HOMO/LUMO gap. For illustration, we have plotted the respective *T*(*E*) functions in Figures S22–S26 and the effect of the Fermi level offset on *S*_mol_ in Figures S27–S31. This suggests that off-resonance charge transport is dominant, in line with the observed electric conductance values, and also that relatively subtle changes in the electronic structure of the junction could move the Fermi level in a way that leads to a switch from HOMO- to LUMO-dominated transport or *vice versa*. This seems to be the case for molecule **4**, where we obtained a small but positive *S*_mol_, while the latter was found to be negative in previous thin-film studies in Au/Pt junctions.^23,24^ Hence, simulations investigating the difference(s) between those two electrode configurations led us to the third conclusion, as outlined below. To this end, we simulated the two experimental setups for molecules **1**, **2,** and **4**, *i.e.,* where both experimental data sets are available. Transmission curves for **1** and **2** with Au/Au and Au/Pt electrodes are similar, even though one difference appears to be that for Au/Pt junctions, the frontier orbitals are downshifted toward lower energies by about 0.2 eV, as shown in Figures S32 and S34, likely reflecting the different electron affinities of the two metals (Au = 223 kJ/mol, Pt = 205 kJ/mol), see Section S3.7 in the Supporting Information. Accordingly, molecules **1** and **2** feature positive *S*_mol_ values in both electrode configurations, as shown in Figures S33 and S35. However, for molecule **4**, the S/Au interaction is *via* a weaker SMe anchor, which does not dictate the electronic structure of the junction in the same way. As a result, the change from Au/Pt to Au/Au substrate electrodes leads to a downward shift of the transmission function relative to *E*_F_, thereby switching charge transport from HOMO-dominated (*S*_mol_ < 0) to LUMO-dominated (*S*_mol_ > 0). Crucially, it appears that the absence of a dominating anchor group allowed for this subtle effect to be observable.

## Conclusions

The present study has revealed a range of new insights into the electric and thermoelectric properties of molecular junctions, where charge transport appears to occur in the off-resonant coherent tunneling regime. We provide a detailed comparison of two methodologies for the measurement of single-molecule charge transport, the well-established STM BJ technique at constant tip/substrate bias, and distance-dependent STM IV spectroscopy, STM IV. To this end, detailed analysis revealed how, under the experimental conditions used, both methods yielded shorter than expected break-off distances compared to the length of the fully extended molecular junction. The exceptions were molecules **1** and **2** in STM BJ experiments, where the measured Δ*z*_mol_ values correspond well with theoretical expectations. While some of the apparent decrease of Δ*z*_mol_ in STM IV spectroscopy may be due to the limited spatial resolution of the measurement (step size: 0.2–0.3 nm), this is not sufficient to explain the observed differences, which are on the order of 1 nm or so. Notably, the applied tip/substrate bias is smaller in STM IV experiments than in STM BJ measurements (±10 *vs* 100 mV), so current- or heating-induced effects are also unlikely to provide a satisfactory explanation. Since the recording of a withdrawal series in STM IV takes somewhat longer than a withdrawal in STM BJ, it is possible that thermal or mechanical drift effects lead to an on-average earlier junction rupture, a hypothesis that would require further systematic study but is beyond the scope of the present work.

Further significant improvements in *G*_mol_ and *S*_mol_ are, however, required to reach more meaningful performance characteristics, which is a reflection of the non-resonant “mid gap” nature of charge transport through the junction. However, our results further support the notion that quantum interference effects can be harnessed to increase *G*_mol_, as observed for molecules **1** and **2**, and potentially also *S*_mol_. Interestingly, we find that for the molecular systems studied here, a strong imbalance between the anchor groups and their interaction with the electrode substrate can lead to a “pinning” effect, where the stronger anchor group effectively dictates the Fermi alignment and hence the nature of the dominating charge carriers. Where such an imbalance is not present, for example, in molecule **4**, subtle differences in bond strength between the anchor and different substrates can lead to a change in Fermi level alignment and a switch from electron to hole transport or *vice versa*. Overall, *S*_mol_ values determined from single-molecule measurements appear to compare well with those extracted from thin-film experiments, where available. This reinforces the important role of single-molecule experiments in identifying structure–function relationships and the optimization of the molecular and interfacial structure.

## Supporting Information Available

Molecular synthesis and characterization; constant bias and temperature-dependent IV experimental procedures; temperature dependence of Δ*z*_mol_ and *G*_mol_; DFT and transport calculations; and determination of optimized structures and binding energies (PDF)
