# Supplementary material for: Quantum Interference and Contact Effects in the Thermoelectric Performance of Anthracene-Based Molecules
Source: J Phys Chem C Nanomater Interfaces. 2023 Apr 10;127(15):7484–91. doi: 10.1021/acs.jpcc.3c00069 (PMC10123663; doi:10.1021/acs.jpcc.3c00069)
Supplement: Supplementary file 1 — jp3c00069_si_001.pdf [file jp3c00069_si_001.pdf]

Quantum Interference and Contact Effects in the Thermoelectric Performance of Anthracene-Based Molecules

Joseph M. Hamill[a*,†], Ali Ismael[b*,†], Alaa Al-Jobory[b,c], Troy L. R. Bennett[d], Maryam Alshahrani [b,e], Xintai Wang[b,f], Maxwell Akers-Douglas[d], Luke A. Wilkinson[d], Benjamin J. Robinson[b], Nicholas J. Long[d], Colin Lambert [b]*, and Tim Albrecht [a]*

Supporting Information

[a]School of Chemistry, University of Birmingham, Edgbaston Campus, Birmingham B15 2TT, United Kingdom.
[b]Physics Department, Lancaster University, Lancaster, LA1 4YB, United Kingdom.
[c]Department of Physics, College of Science, University of Anbar, Anbar, Iraq.
[d]Department of Chemistry, Imperial College London, MSRH, White City, London, W12 0BZ, United Kingdom.
[e]Physics Department, College of Science, University of Bisha, Bisha 61922, P.O Box 344, Kingdom of Saudi Arabia.
[f]School of Information Science and Technology, Dalian Maritime University, Dalian, China.

†These authors contributed equally to this work.

*To whom correspondence should be addressed. e-mail: jmh@chem.ku.dk; k.ismael@lancaster.ac.uk; c.lambert@lancaster.ac.uk; t.albrecht@bham.ac.uk

1. Molecular Synthesis

1.1 Materials and Methods

All reactions were performed with the use of standard air-sensitive chemistry and Schlenk line techniques, under an atmosphere of nitrogen. No special precautions were taken to exclude air during any work-ups. All commercially available reagents were used as received from suppliers, without further purification. 4-(Ethynyl)phenyl-thiopropionitrile was synthesised through an adapted literature procedure.[1] Solvents used in reactions were collected from solvent towers sparged with nitrogen and dried with 3 Å molecular sieves, apart from DIPA, which was distilled onto activated 3 Å molecular sieves under nitrogen. We have previously reported the synthesis of compounds (**1**-**5**).[2,3] Sonogashira couplings reported within this work were performed following a previously established method.[3,4]

Instrumentation. ^1H and $^{13}C\{^1H\}$ NMR spectra were recorded on a Bruker Avance 400 MHz spectrometer and referenced to the residual solvent peaks of $CDCl_3$ at 7.26 and 77.16 ppm, respectively. Coupling constants are measured in Hz. Mass spectrometry analyses were conducted by Dr. Lisa Haigh of the Mass Spectrometry Service, Imperial College London.

1.2 Synthesis

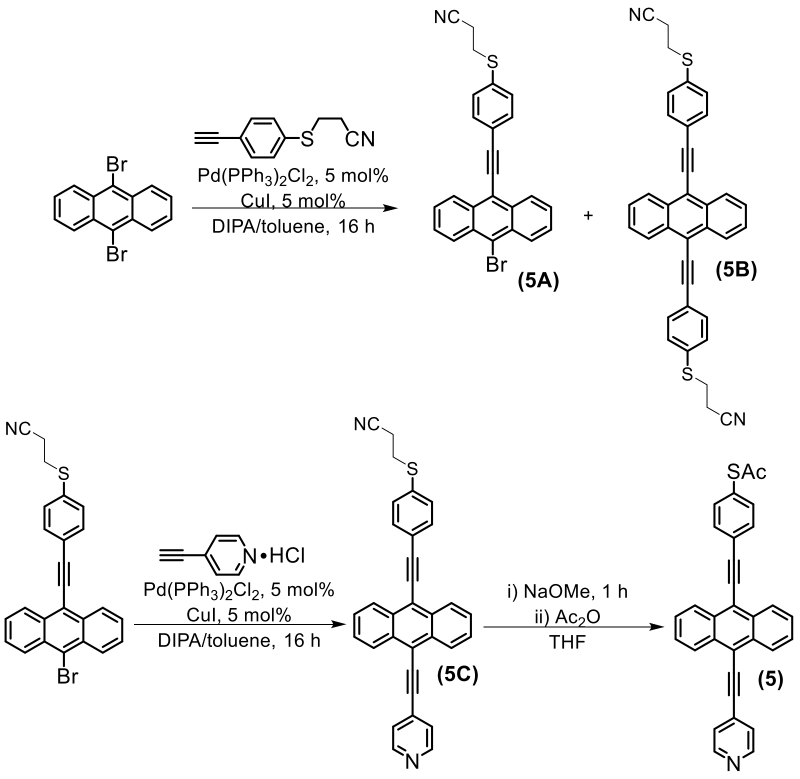

Scheme S1: Reaction pathway utilised to synthesise molecule **5**.

9-Bromo-10-(4-(ethynyl)phenylthiocyanoethyl)anthracene (8A)

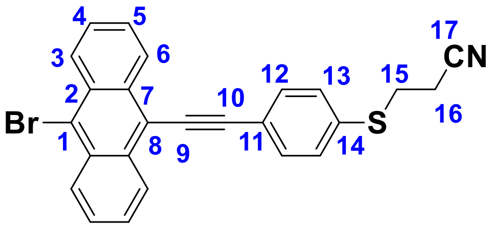

9,10-Dibromoanthracene (3.60 g, 10.72 mmol), 4-(ethynyl)phenylthiocyanoethyl (0.51 g, 2.71 mmol) and CuI (0.06 g, 0.29 mmol) were dissolved in DIPA (10 mL) and toluene (250 mL). $Pd(PPh_3)_2Cl_2$ (0.02 g, 0.33 mmol) was added and the mixture was stirred at 50°C for >16 hours to give a dark green solution. The

solvent was removed *in vacuo* before the crude product was dissolved in DCM, filtered and subsequently purified by chromatography on an alumina V column, eluting with n-hexane/DCM (1:0 → 4:1) to give the product as a bright orange solid (0.52 g, 1.18 mmol, 43%).

^{1}H NMR ($CDCl_3$, 298 K, 400 MHz): **δ_H** = 8.65-8.60 (m, 2H, *H*6), 8.58-8.53 (m, 2H, *H*3), 7.70 (dt, $^{3}J_{H\text{-}H}$ = 8.8, $^{4}J_{H\text{-}H}$ = 2.0 Hz, 2H, *H*13), 7.66-7.59 (m, 4H, *H*4, *H*5), 7.44 (dt, $^{3}J_{H\text{-}H}$ = 8.8, $^{4}J_{H\text{-}H}$ = 2.0 Hz, 2H, *H*12), 3.21 (t, $^{3}J_{H\text{-}H}$ = 7.6 Hz, 2H, *H*15), 2.67 (t, $^{3}J_{H\text{-}H}$ = 7.6 Hz, 2H, *H*16) ppm; **^{13}C{^{1}H} NMR** ($CDCl_3$, 298 K, 100 MHz): **δ_C** = 134.6 (Ar-C-C), 133.1 (Ar-C-C), 132.5 (Ar-C-H), 130.6 (Ar-C-H), 130.4 (Ar-C-C), 128.4 (Ar-C-H), 127.6 (Ar-C-H), 127.2 (Ar-C-H), 127.1 (Ar-C-H), 124.7 (Ar-C-C), 122.6 (Ar-C-C), 118.0 (Ar-C-C), 117.9 (Ar-C-C), 101.0 (-C≡C-), 87.4 (-C≡C-), 29.9 (-CH_2-), 18.4 (-CH_2-) ppm; **MS APCI**: calcd. [M]+ 442.0260; found. 442.0272.

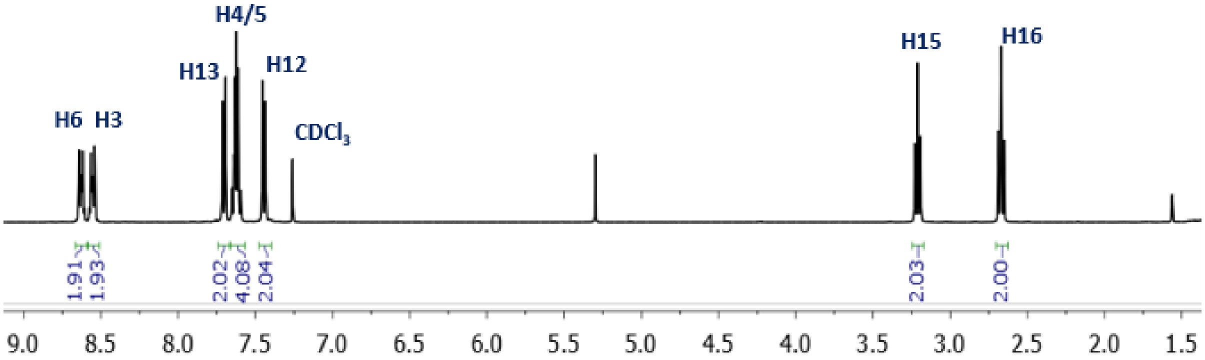

Fig. S1: The ^{1}H NMR spectrum of **5A** in $CDCl_3$.

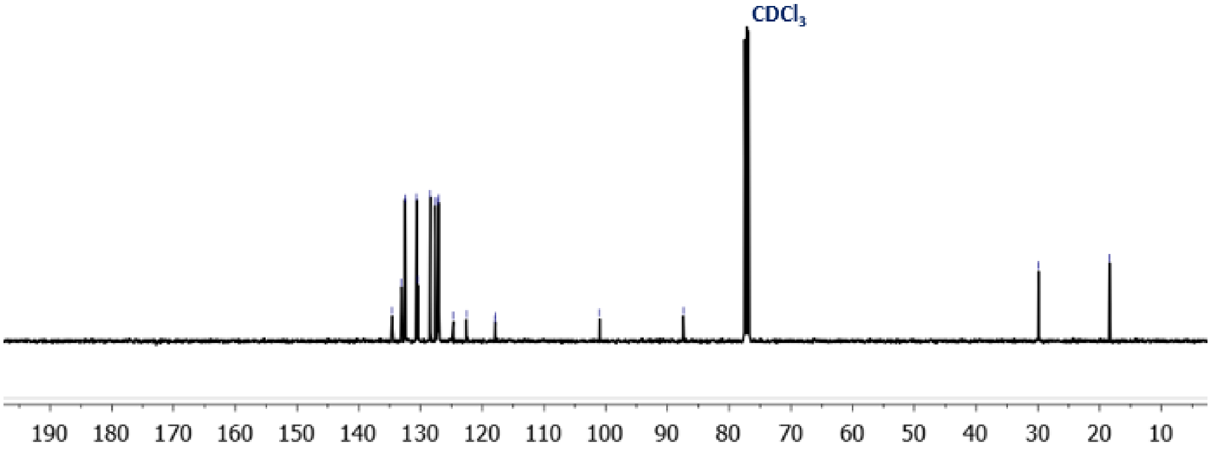

Fig. S2: The ^{13}C{^{1}H} NMR spectrum of **5A** in $CDCl_3$.

9,10-Di(4-(ethynyl)phenylthiocyanoethyl)anthracene (8B)

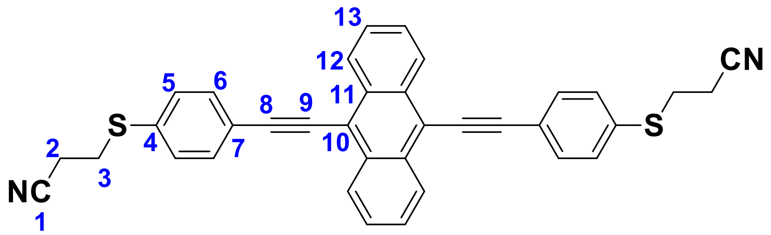

9,10-Dibromoanthracene (3.60 g, 10.72 mmol), 4-(ethynyl)phenylthiocyanoethyl (0.51 g, 2.71 mmol) and CuI (0.06 g, 0.29 mmol) were dissolved in DIPA (10 mL) and toluene (250 mL). $Pd(PPh_3)_2Cl_2$ (0.02 g, 0.33 mmol) was added and the mixture was stirred at 50°C for >16 hours to give a dark green solution. The solvent was removed *in vacuo* before the crude product was dissolved in DCM, filtered and subsequently purified by chromatography on an alumina V column, eluting with n-hexane/ethyl acetate (1:0 → 0:1). This compound was further purified through recrystallization from ethyl acetate to give a bright orange solid (0.26 g, 0.49 mmol, 18%, <5% impurity).

^{1}H NMR ($CDCl_3$, 298 K, 400 MHz): **δ_H** = 8.69-8.64 (m, 4H, *H*12), 7.74 (dt, $^3J_{H-H}$ = 8.8, $^4J_{H-H}$ = 2.0 Hz, 2H, *H*5), 7.68-7.63 (m, 4H, *H*13), 7.47 (dt, $^3J_{H-H}$ = 8.8, $^4J_{H-H}$ = 2.0 Hz, 2H, *H*6), 3.21 (t, $^3J_{H-H}$ = 7.6 Hz, 2H, *H*3), 2.67 (t, $^3J_{H-H}$ = 7.6 Hz, 2H, *H*2) ppm; **^{13}C{^{1}H} NMR** ($CDCl_3$, 298 K, 100 MHz): **δ_C** = 134.6 (Ar-C-C), 132.6 (Ar-C-H), 132.2 (Ar-C-C), 130.6 (Ar-C-H), 127.3 (Ar-C-H), 127.1 (Ar-C-H), 122.6 (Ar-C-C), 118.5 (Ar-C-C), 117.9 (Ar-C-C), 101.8 (-C≡C-), 87.9 (-C≡C-), 29.9 (-CH_2-), 18.4 (-CH_2-) ppm; **MS APCI**: calcd. [M]+ 549.1454; found. 549.1474.

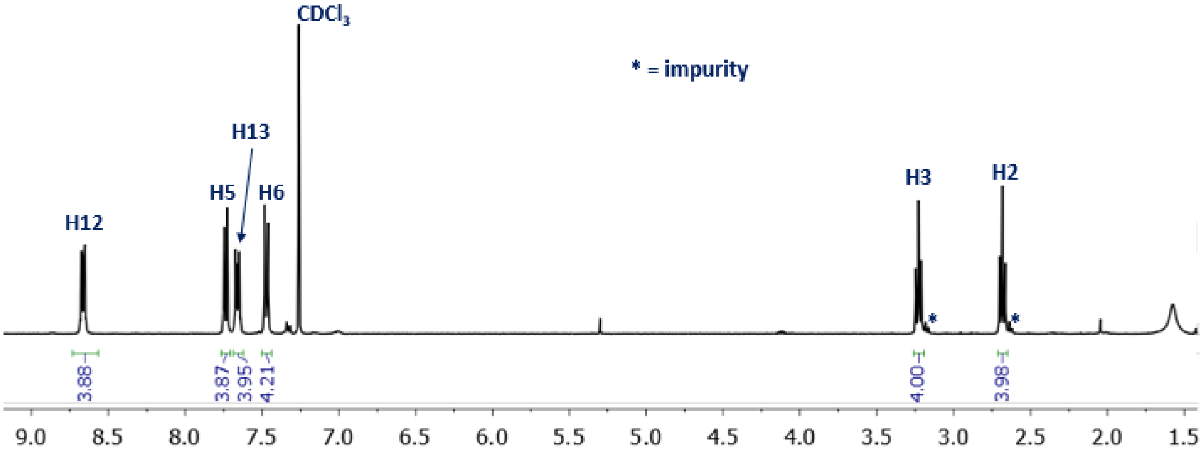

Fig. S3: The ^{1}H NMR spectrum of **5B** in $CDCl_3$.

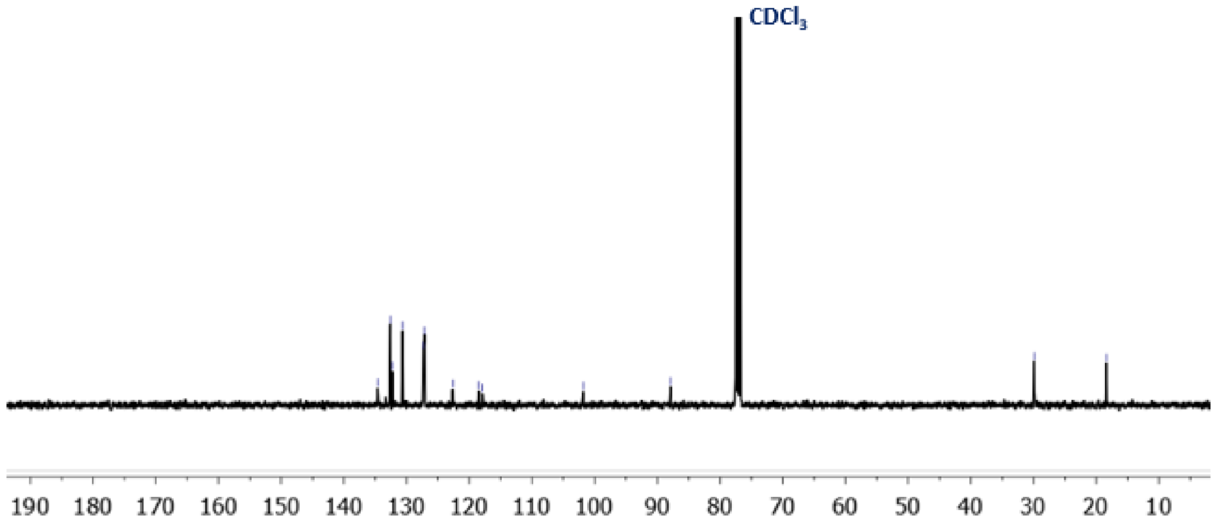

Fig. S4: The $^{13}C\{^{1}H\}$ NMR spectrum of **5B** in $CDCl_3$.

9-(4-(Ethynyl)pyridine)-10-(4-(ethynyl)phenylthiocyanoethyl)anthracene (5C)

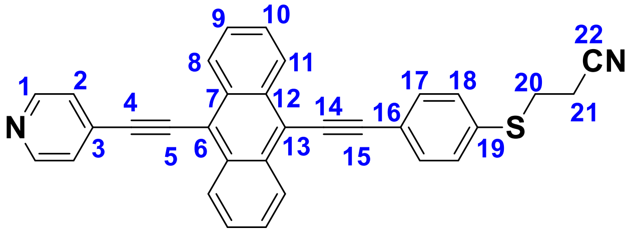

5A (0.26 g, 0.59 mmol), 4-(ethynyl)pyridine hydrochloride (0.18 g, 1.26 mmol) and CuI (0.04 g, 0.43 mmol) were dissolved in DIPA (10 mL) and DMF (150 mL). $Pd(PPh_3)_2Cl_2$ (0.05 g, 0.06 mmol) was added and the mixture was stirred at 75°C for >16 hours to give a dark red solution. The solution was diluted with ethyl acetate (300 mL) and washed with water (3 x 150 mL) before being dried over $MgSO_4$ and filtered. The solvent was removed *in vacuo* to give a bright orange solid that was purified by chromatography on an alumina V column, eluting with n-hexane/DCM (1:1 → 0:1), before being washed with copious amounts of hexane to give the final product as a bright orange solid (0.07 g, 0.15 mmol, 26%).

^{1}H NMR ($CDCl_3$, 298 K, 400 MHz): **δ_H** = 8.72 (br s, 2H, *H*1), 8.68-8.59 (m, 4H, *H*8, *H*11), 7.73 (dt, $^{3}J_{H-H}$ = 8.8, $^{4}J_{H-H}$ = 2.0 Hz, 2H, *H*18), 7.70-7.63 (m, 4H, *H*9, *H*10), 7.62 (d, $^{3}J_{H-H}$ = 5.2 Hz, 2H, *H*2), 7.47 (dt, $^{3}J_{H-H}$ = 8.8, $^{4}J_{H-H}$ = 2.0 Hz, 2H, *H*17), 3.23 (t, $^{3}J_{H-H}$ = 7.6 Hz, 2H, *H*20), 2.68 (t, $^{3}J_{H-H}$ = 7.6 Hz, 2H, *H*21) ppm; **$^{13}C\{^{1}H\}$ NMR** ($CDCl_3$, 298 K, 100 MHz): **δ_C** = 150.1 (Ar-C̲-H), 134.8 (Ar-C̲-C), 132.6 (Ar-C̲-H), 132.5 (Ar-C̲-C), 132.1 (Ar-C̲-C), 131.5 (Ar-C̲-C), 130.6 (Ar-C̲-H), 127.5 (Ar-C̲-H), 127.4 (Ar-C̲-H), 127.2 (Ar-C̲-H), 127.0 (Ar-C̲-H), 125.6 (Ar-

C-H), 122.4 (Ar-C-C), 119.6 (Ar-C-C), 117.9 (Ar-C-C), 117.2 (Ar-C-C), 102.3 (-C≡C-), 99.5 (-C≡C-), 91.0 (-C≡C-), 87.7 (-C≡C-), 29.9 (-CH_2-), 18.4 (-CH_2-) ppm; **MS APCI**: calcd. [M]+ 465.1420; found. 465.1420.

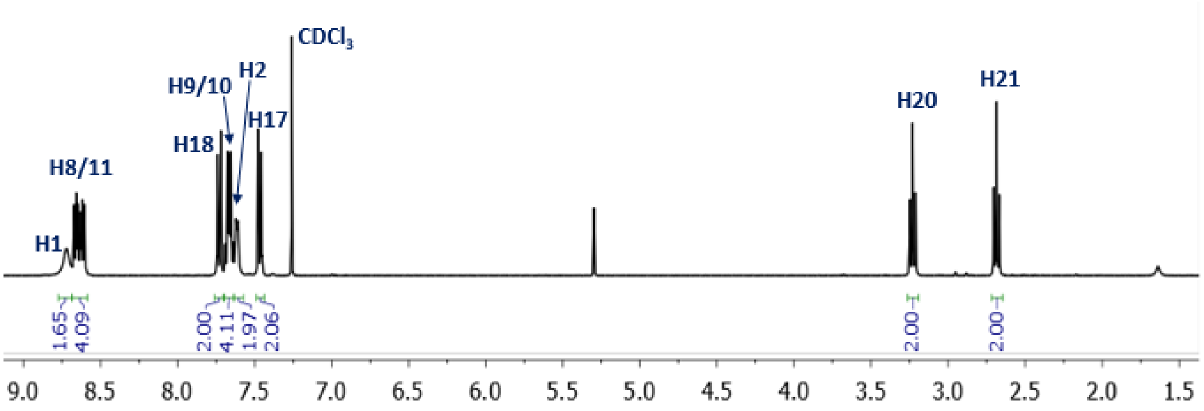

Fig. S5: The ^{1}H NMR spectrum of **5C** in $CDCl_3$.

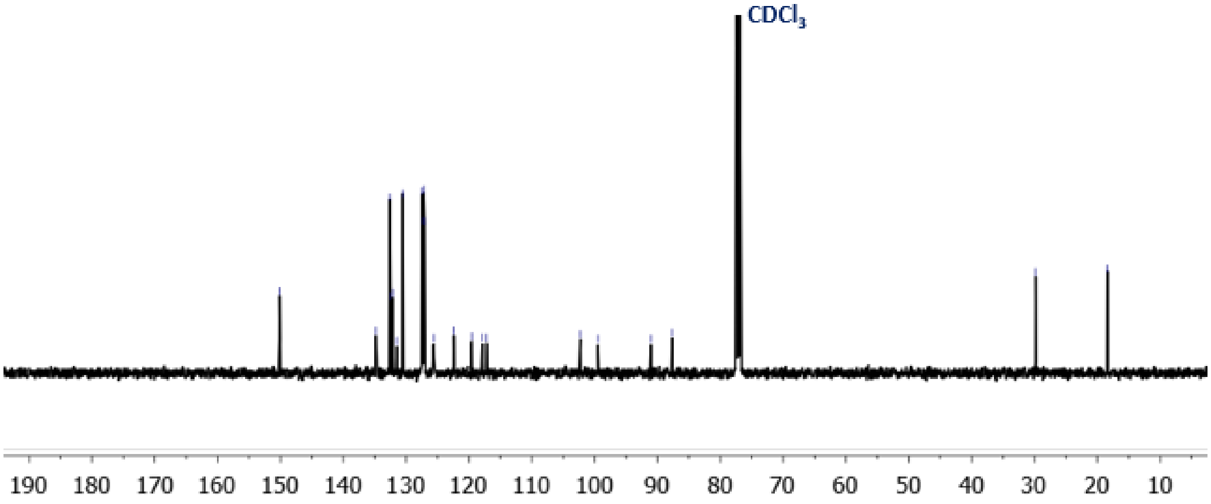

Fig. S6: The $^{13}C\{^{1}H\}$ NMR spectrum of **8c** in $CDCl_3$.

9-(4-(Ethynyl)pyridine)-10-(4-(ethynyl)phenylthioacetate)anthracene (5)

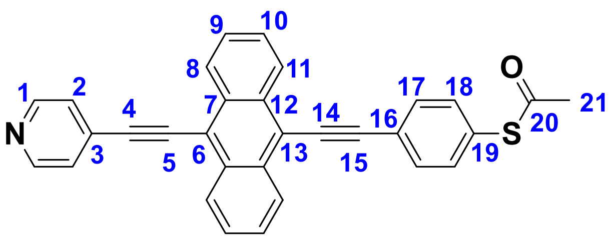

5C (0.06 g, 0.13 mmol) and NaOMe (0.25 g, 0.02 mmol) were dissolved in THF (20 mL) and stirred at room temperature for 1 hour to give a dark red solution. Acetic anhydride (1 M in THF, 0.50 mL) was added dropwise and the solution was left to stir for 30 minutes. The solvent was removed *in vacuo* and the crude product was dissolved in toluene (300 mL), washed with water (3 x 100 mL) and brine (100 mL),dried over $MgSO_4$ and filtered. The solvent was removed *in vacuo* to give the product as a bright orange solid (0.03 g, 0.07 mmol, 51%).

^{1}H NMR ($CDCl_3$, 298 K, 400 MHz): **δ_H** = 8.72 (d, $^3J_{H\text{-}H}$ = 5.2 Hz, *H*1), 8.70-8.60 (m, 4H, *H*8, *H*11), 7.81 (dt, $^3J_{H\text{-}H}$ = 8.8, $^4J_{H\text{-}H}$ = 2.0 Hz, 2H, *H*18), 7.72-7.63 (m, 4H, *H*9, *H*10), 7.62 (dd, $^3J_{H\text{-}H}$ = 5.2, $^4J_{H\text{-}H}$ = 1.6 Hz, *H*2), 7.51 (dt, $^3J_{H\text{-}H}$ = 8.8, $^4J_{H\text{-}H}$ = 2.0 Hz, 2H, *H*17), 2.48 (s, 3H, *H*21) ppm; **^{13}C{^{1}H} NMR** ($CDCl_3$, 298 K, 100 MHz): **δ_C** = 193.5 (-C=O), 150.1 (Ar-C-H), 134.6 (Ar-C-H), 132.5 (Ar-C-C), 132.4 (Ar-C-H), 132.2 (Ar-C-C), 131.5 (Ar-C-C), 129.0 (Ar-C-C), 127.5 (Ar-C-H), 127.5 (Ar-C-H), 127.3 (Ar-C-H), 127.0 (Ar-C-H), 125.6 (Ar-C-H), 124.5 (Ar-C-C), 119.5 (Ar-C-C), 117.3 (Ar-C-C), 102.3 (-C≡C-), 99.5 (-C≡C-), 91.1 (-C≡C-), 88.0 (-C≡C-), 30.5 (-CH_3) ppm; **MS APCI**: calcd. [M]+ 454.1260; found. 454.1241.

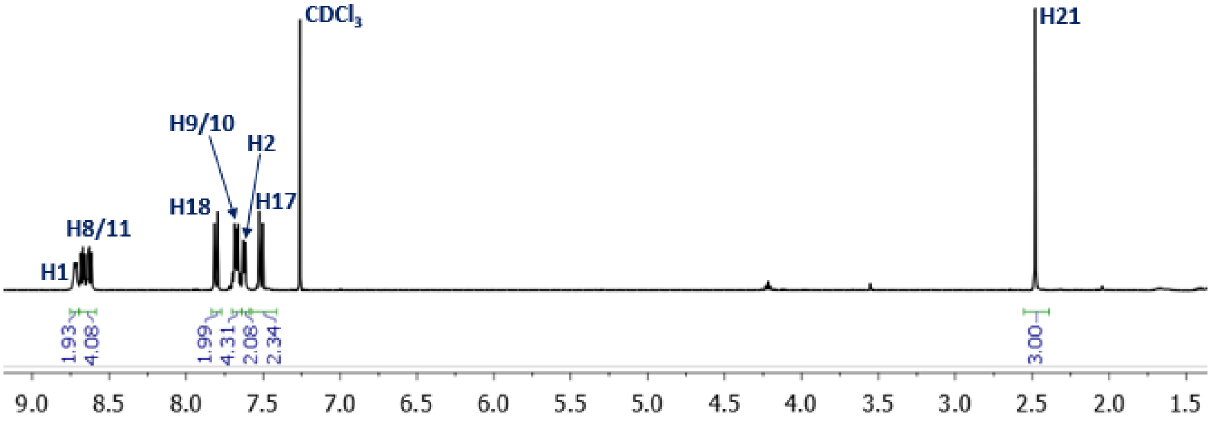

Fig. S7: The ^{1}H NMR spectrum of **5** in $CDCl_3$.

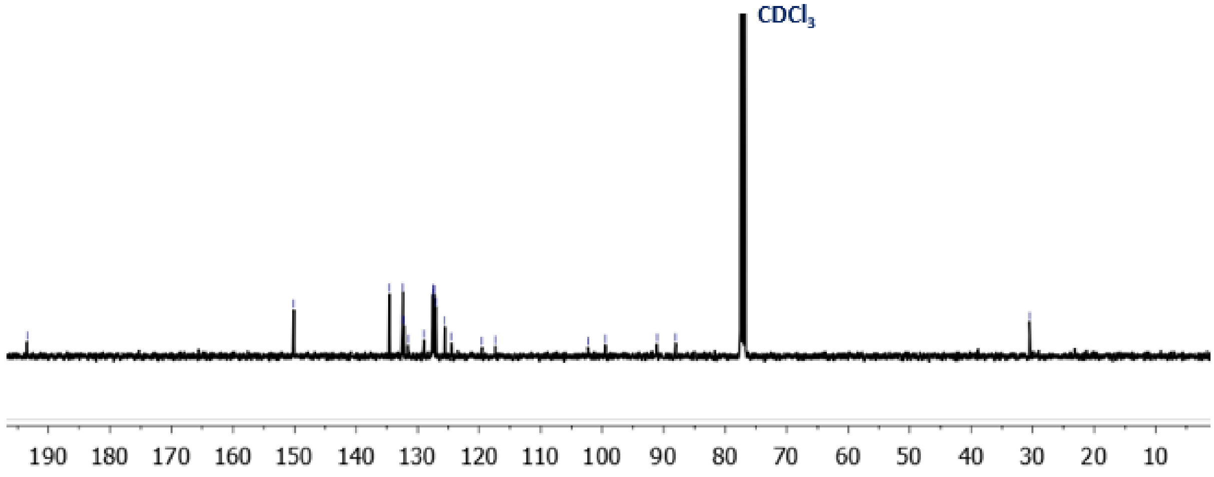

Fig. S8: The $^{13}C\{^{1}H\}$ NMR spectrum of **5** in $CDCl_3$.

2. Experimental Methods: tunnelling spectroscopy

2.1 STM BJ IV results for Au, and Molecules **1**, **4**, and **5**

As a measure of the background, without molecules present, Figs. S1 (a) –(c) show results obtained when no molecular adlayer was present on the Au substrate. Acquisition of data followed the same procedures, whereby a thermal gradient was applied and STM BJ IV measurements were performed obtaining results similar to Figs. 3(a) – (d) in the main text. Without a molecule present, sub-G_0 I/V sweeps were fewer in number, and occurred at shorter *Δz*. Nonetheless, a cluster similar to the green cluster in Figs. (c) and (d) yielded distributions of *ΔV*, *G*, and *Δz* values for different *ΔT*. These were plotted in Figs. S1(a), (b), and (c), respectively. Linear fits for each variable across all *ΔT* were also plotted in black. The slope of *ΔV* vs *ΔT* determined a background Au/gap/Au thermopower, -0.2(2) $\mu V\ K^{-1}$, of our method and analysis (see Table 1). The slopes and standard errors on the slope for both *G* and *Δz* are summarized in Table S1. A complete discussion of dependences on thermal gradient can be found in section S2.5.

Complete results for molecule **2** are presented in section 2.2 and Fig. 4 of the main text. Results for molecules **1**, **4**, and **5** are plotted in Figs. S9(d)-(f) and S10. Replicate 1 (blue), 2 (red), and (when present) 3 (green) were plotted and fit to separate linear fits (navy, crimson, and dark green, respectively). As with molecule **5**, the single-molecule Seebeck coefficients, calculated from the slopes of the combined *ΔV* vs *ΔT* distributions, are plotted with black lines, as summarized in Table 1.

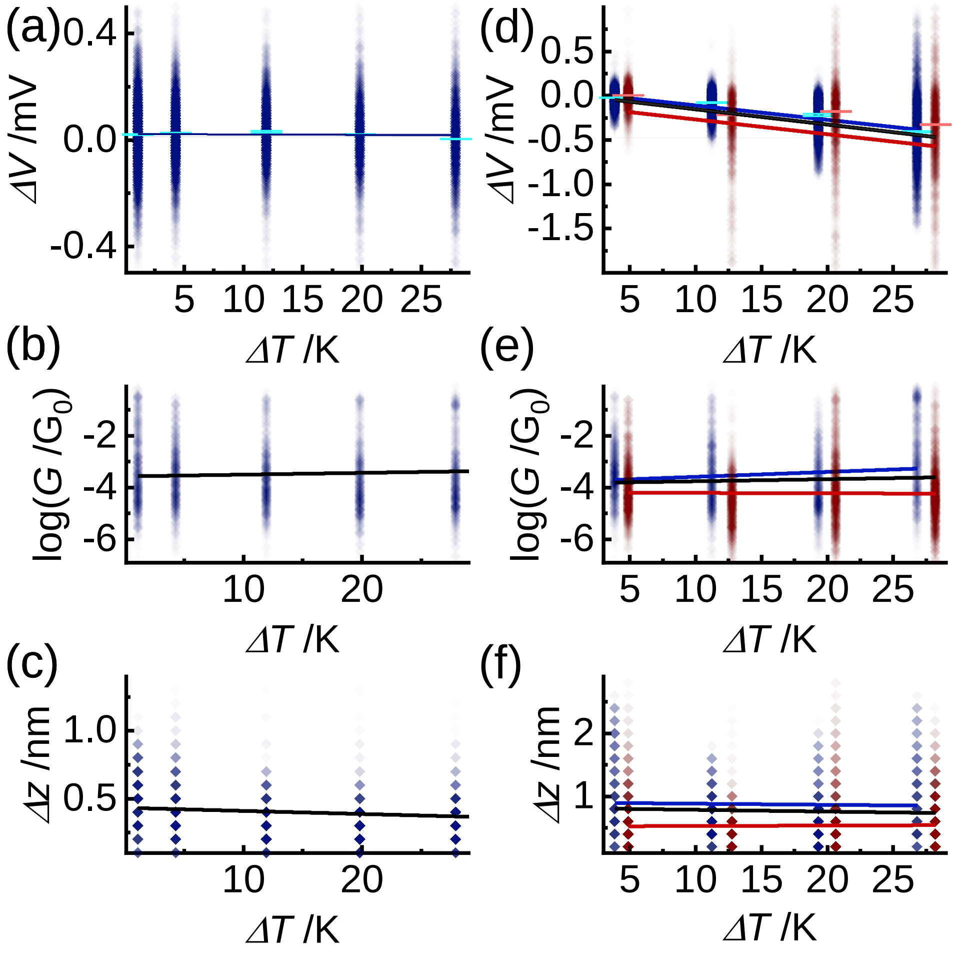

FIG S9. Scatter plots of ΔV vs ΔT measurements of (a) **Au/Au** with trend line in black, and (d) **1** with separate trend lines for two separate experiments (red and blue), and combined trend lines with 95% confidence intervals (black). Scatter plots of G vs ΔT measurements of (b) **Au/Au** with trend line in black, and (e) molecule **1** with separate trend lines for two separate experiments (red and blue), and combined trend lines with 95% confidence intervals (black). Scatter plots of Δz vs ΔT measurements of (c) **Au/Au** with trend line in black, and (f) molecule **1** with separate trend lines for two separate experiments (red and blue), and combined trend lines with 95% confidence intervals (black). Light blue and red cross hairs are mean and standard error of individual distributions, included as aids for the eye.

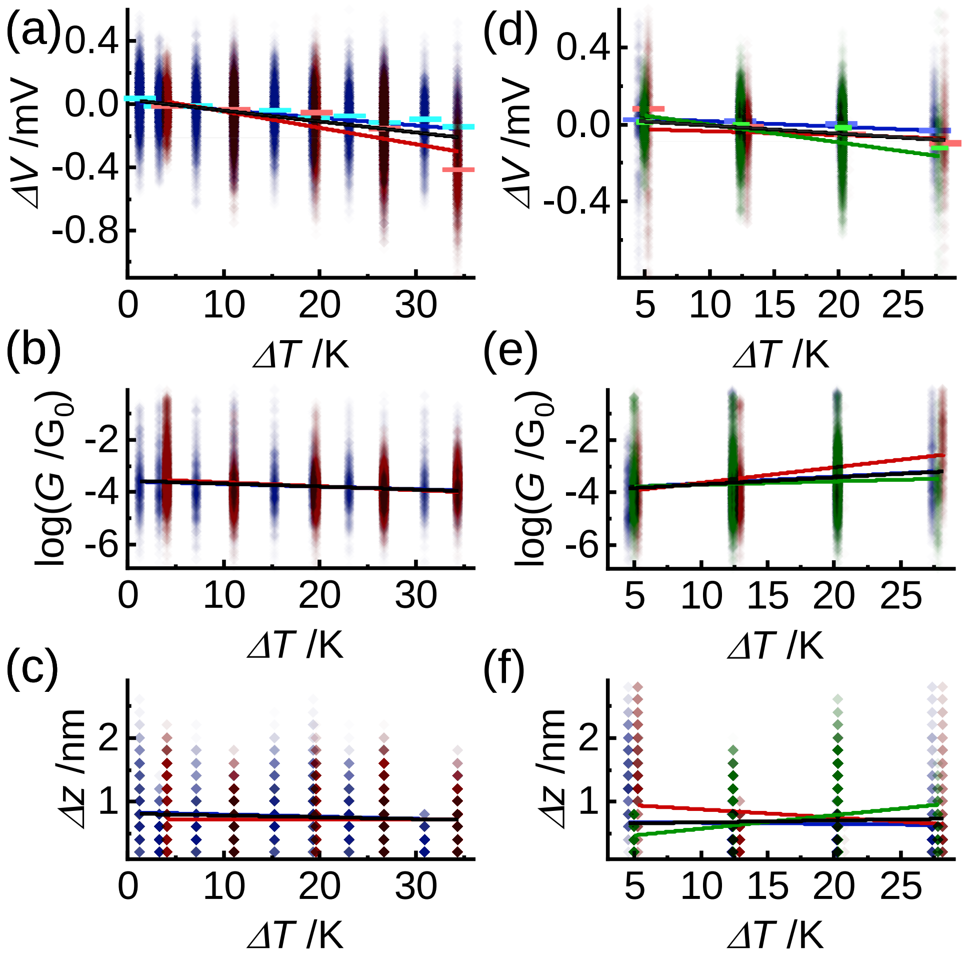

FIG S10. Scatter plots of *ΔV* vs *ΔT* measurements of (a) molecules **4** and (d) **5** with separate trend lines for two (for molecule **4**; three for molecule **5**) separate experiments (red and blue and green), and combined trend lines with 95% confidence intervals (black). Scatter plots of *G* vs *ΔT* measurements of (b) molecules **4** and (e) **5** with separate trend lines for two separate experiments (red and blue), and combined trend lines with 95% confidence intervals (black). Scatter plots of *Δz* vs *ΔT* measurements of (c) **4** and (f) **5** with separate trend lines for two separate experiments (red and blue), and combined trend lines with 95% confidence intervals (black). Light blue and red and green cross hairs are mean and standard error of individual distributions, included as aids for the eye.

2.2 Constant bias single-molecule break junctions

Constant bias results for molecules **1**, **3**-**5** are found in Figs. S11-S14. Constant bias results served to confirm the reliability of the molecule in the junction, and the obtain values for G_{mol} and Δz_{mol} to validate the STM BJ IV results. Figs. S11-S14 were created following the same procedures outlined in section 2.3 in the main text. Notably, molecule **3** was successfully measured in STM BJ mode, but not in STM BJ IV mode, despite an intense effort. At present, the reason for this difference in behaviour is unclear, cf. main text.

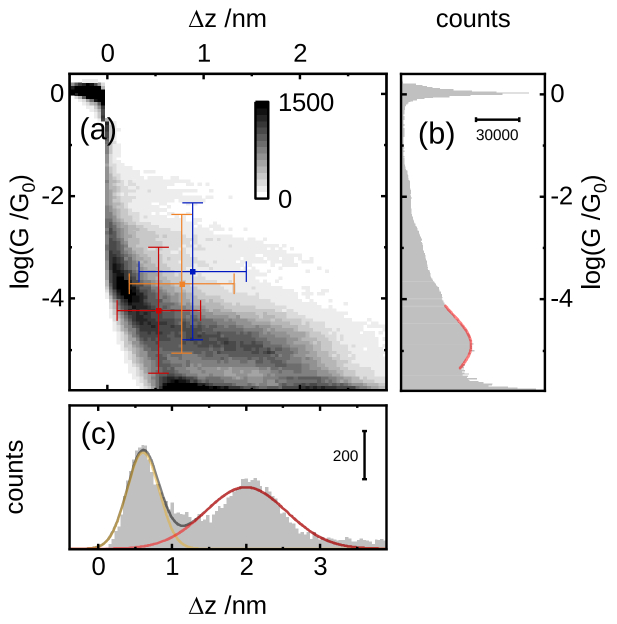

FIG S11. Example constant bias results for molecule **1** with c. 17k traces. (a) 2D conductance-displacement intensity plot of all traces. Cross hairs are trial 1 (blue) and trial 2 (red) and combined (orange) mean and standard deviation values of molecular cluster from STM BJ IV measurements. (b) 1D conductance histogram of all data in (a), with Gaussian peak fit (red). (c) Break-off distance histogram, determined at conductance $10^{-5.4}$ G_0, with two peak fit to separate tunnelling (gold) and molecular (red) trace displacements. Junction formation probability was 62%.

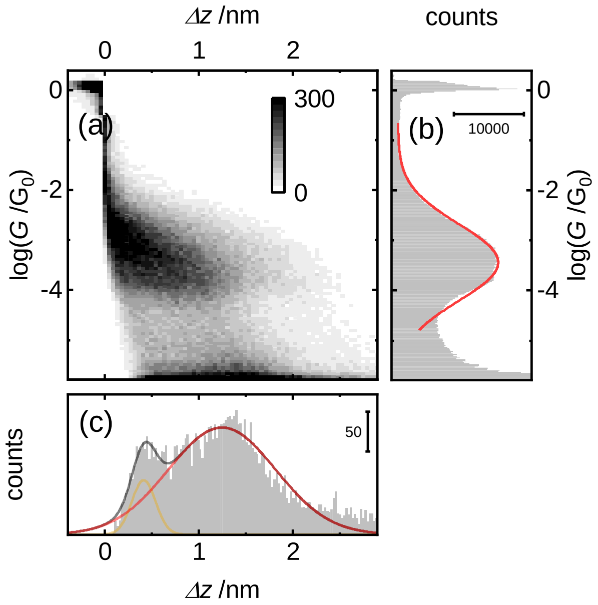

FIG S12. Example constant bias results for molecule **3**. (a) 2D conductance-displacement intensity plot of all traces. (b) 1D conductance histogram of all data in (a), with Gaussian peak fit (red). (c) Break-off distance histogram, determined at conductance $10^{-5.2}$ G_0, with 2 peak fit to separate tunnelling (yellow) and molecular (red) trace displacements. Junction formation probability was 90%.

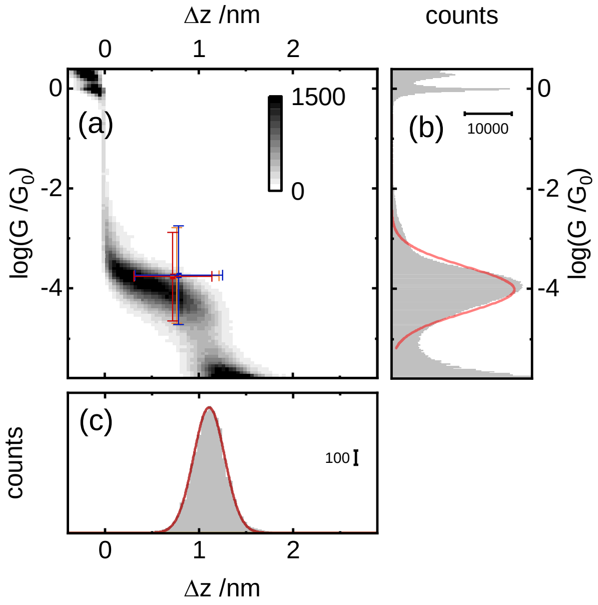

FIG S13. Example constant bias results for molecule **4**. (a) 2D conductance-displacement intensity plot of all traces. Cross hairs are trial 1 (blue), trial 2 (red), and combined (orange) mean and standard deviation values of molecular cluster from STM BJ IV measurements. (b) 1D conductance histogram of all data in (a), with Gaussian peak fit (red). (c) Break-off distance histogram, determined at conductance $10^{-5.2}$ G_0, with peak fit (red). Junction formation probability was close to 100%.

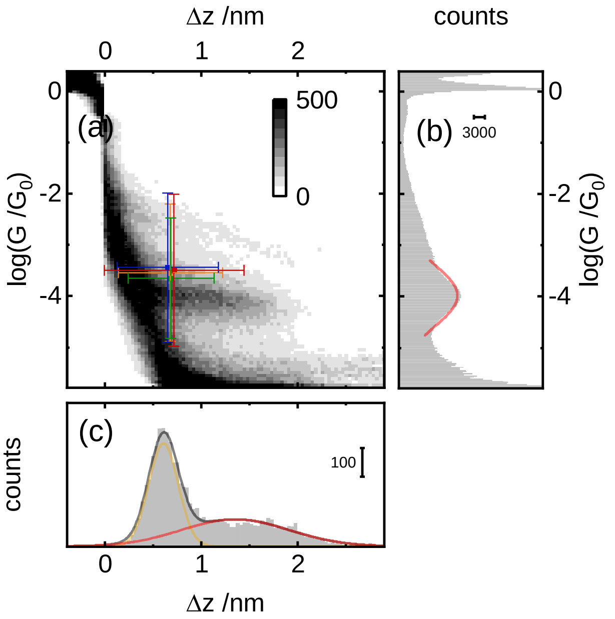

FIG S14. Example constant bias results for molecule **5**. (a) 2D conductance-displacement intensity plot of all traces. Cross hairs are trial 1 (blue), trial 2 (red), and trial 3 (green), and combined (orange) mean and standard deviation values of molecular cluster from STM BJ IV measurements. (b) 1D conductance histogram of all data in (a), with Gaussian peak fit (red). (c) Break-off distance histogram, determined at conductance $10^{-5.2}$ G_0, with 2 peak fit to separate tunnelling (yellow) and molecular (red) trace displacements. Junction formation probability was 49%.

2.3 Temperature dependence of Δz_{mol} and G_{mol}

Table S1 summarizes the slopes and standard errors for Δz and G. The expected charge transport regime for all molecules measured in this study was coherent transport. As a consequence, we expect there to be no (significant) dependence of molecular conductance on the applied temperature gradient. This is indeed borne out in the data. Likewise, there does not seem to be a systematic and statistically significant dependence of the molecular break-off distance on ΔT, which might suggest that the junction stability is not affected the change in temperature gradient.

Table S1. Summary of temperature dependencies.

	**Δz**	*error*	**G**	*error*
Molecule	*Slope* nm K^{-1}	*Std Err* nm K^{-1}	*Slope* $\log(G\,G_0^{-1})\,K^{-1}$	*Std Err* $\log(G\,G_0^{-1})\,K^{-1}$
**1** (1,5 SAc2)	-2.8E-3	4.1E-4	8.2E-3	1.0E-3
**2** (9,10 SAc2)	7.1E-3	3.8E-4	1.3E-2	6.5E-4
3 *(*9,10 SMe2*)*	---	---	---	---
**4** (9,10 SMe,N)	-2.8E-3	1.8E-4	-1.1E-2	3.8E-4
**5** (9,10 SAc,N)	3.1E-3	4.7E-4	2.7E-2	1.1E-3
Au	-2.2E-3	1.0E-4	6.8E-3	6.4E-2

3. Theoretical details

3.1 DFT and Transport Calculations

The ground state Hamiltonian and optimized geometry of each molecule was obtained using the density functional theory (DFT) code SIESTA.[5,6] The local density approximation (LDA) exchange correlation functional was used along with double zeta polarized (DZP) basis sets and the norm conserving pseudo potentials. The real space grid was defined by a plane wave cut-off of 250 Ry. Geometry optimization was carried out to a force tolerance of 0.01 eV/Å. This process was repeated for a unit cell with the molecule located between gold electrodes, in which the optimized distances between Au and the anchor groups were obtained. From the ground state Hamiltonian, the transmission coefficient, the room temperature electrical conductance G and Seebeck coefficient S were obtained using the GOLLUM quantum transport code.[7]

3.2 Optimised DFT Structures of Isolated Molecules

Fig S15 shows the optimum geometries of the isolated molecules **1-5**, obtained by using the local density approximation (LDA). We also computed results using GGA and found that the resulting transmission functions were comparable with those obtained using LDA.[8,9,10] Molecules **1-4** of the 5 molecules were studied previously.[2]

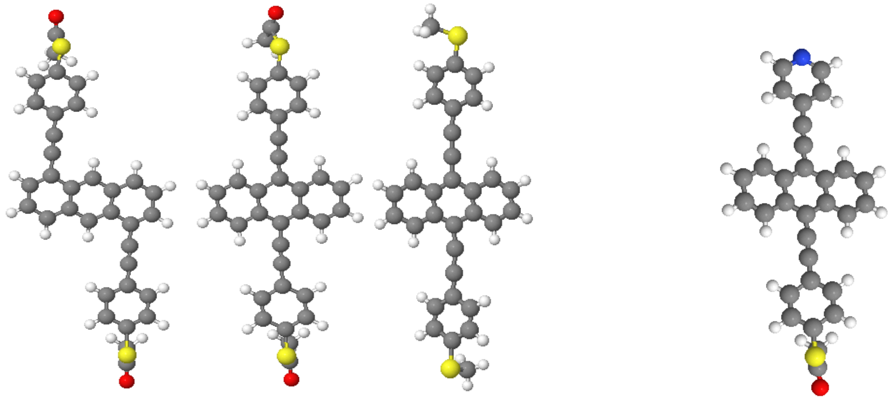

1 2 3 4 5

FIG S15. Fully relaxed isolated molecules **1-5 (1-4** previously reported[2]**)**. Key: C = grey, H = white, O = red, S = yellow, N = blue.

3.3 Binding energy of molecules on Au

To calculate the optimum binding distance between N/S/SMe anchor groups and Au(111) surfaces, we used DFT and the counterpoise method, which removes basis set superposition errors (BSSE). The binding distance is defined as the distance between the gold surface and the N/S/SMe terminus of the anchor groups. Here, the molecule is defined to be entity A and the gold electrode to be entity B. The ground state energy of the total system is calculated using SIESTA and is denoted $\mathrm{E}_{\mathrm{AB}}^{\mathrm{AB}}$. The energy of each entity is then calculated in a fixed basis, which is achieved using ghost atoms in SIESTA. Hence, the energy of A in the presence of the fixed basis is defined as $\mathrm{E}_{\mathrm{A}}^{\mathrm{AB}}$ and for the gold as $\mathrm{E}_{\mathrm{B}}^{\mathrm{AB}}$. The binding energy is then calculated using the following equation:

$$\text{Binding Energy} = \mathrm{E}_{\mathrm{AB}}^{\mathrm{AB}} - \mathrm{E}_{\mathrm{A}}^{\mathrm{AB}} - \mathrm{E}_{\mathrm{B}}^{\mathrm{AB}} \qquad \text{(S1)}$$

We then considered the nature of the binding depending on the gold surface structure. We calculated the binding to a Au pyramid on a surface with the N/S/SMe atom/s binding at a ‘top’ site and then varied the binding distance. Figure S16 (left) shows that value of distances = 2.3, 2.4 and 2.7 Å give the optimum distances, at approximately 0.5, 0.8 and 0.4 eV (N/S/SMe respectively).

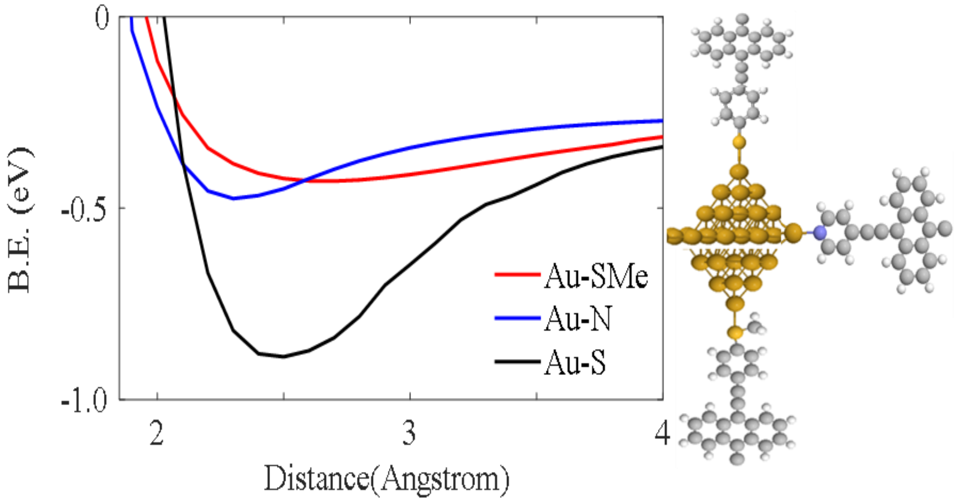

FIG S16. Example of a binding energy plot for three different anchors Au-SMe, Au-N and Au-S (left), with an idealised adatom configuration at the Au lead interface (right, Au-S, Au-N and Au-SMe). Key: C = grey, H = white, N = blue, S = light yellow, Au = dark yellow.

3.4 Optimised DFT Structures of Compounds within Junctions

We considered the most energetically favourable binding configuration, since this corresponds to the most probable molecular junction. After calculating the optimum geometry of the isolated molecular wire, the acetate atoms were removed from the terminal thioacetate groups and the molecule was attached to gold electrodes. Finally, the geometry of the whole junction was further relaxed with fixed Au electrodes. We again employed the SIESTA code to calculate self-consistent optimised geometries, ground state Hamiltonians and overlap matrix elements for each metal-molecule-metal junction. Leads were modelled as 625 atom slabs, terminated with 11-atom Au(111) tips. The optimised structures were then used to compute the transmission curve for each compound. The DFT optimised geometries **1-5** are shown here, in Figures S17-S21. Key: C = grey, H = white, S = light yellow, N = blue, Au = dark yellow.

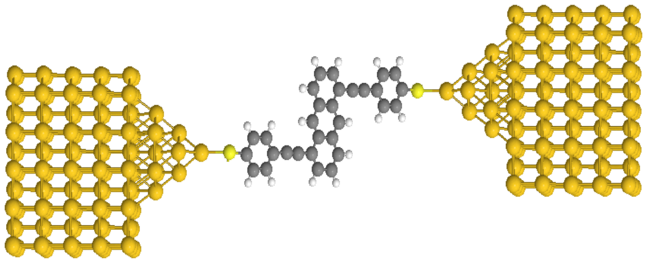

FIG S17. Optimised structure of **1,** (1,5-Di(4-(ethynylphenyl)thioacetate) anthracene).

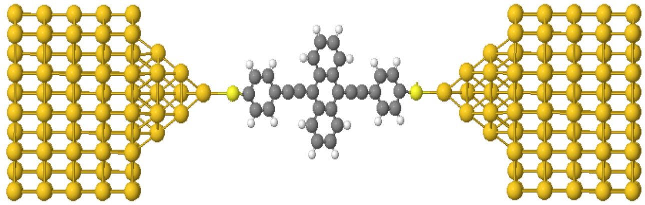

FIG S18. Optimised structure of **2**, (9,10-Di(4-(ethynylphenyl)thioacetate)anthracene).

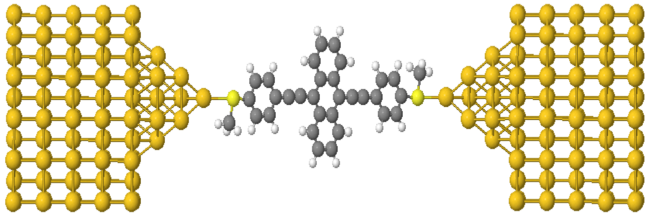

FIG S19. Optimised structure of **3**, (9,10-Di(4-ethynylthioanisole)anthracene).

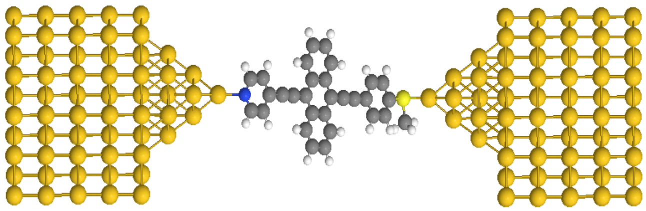

FIG S20. Optimised structure of **4**, (9-(4-(ethynylphenyl)thioacetate)-10-(4-ethynylpyridine)anthracene).

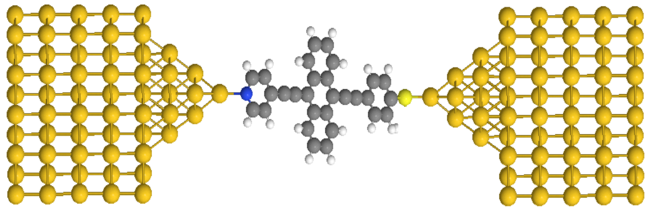

FIG S21. Optimised structure of **5**, (9-(4-ethynlthioanisole)-10-(4-ethynylpyridine)anthracene).

The transmission coefficient curves $T(E)$, obtained from using the GOLLUM transport code, were calculated for the five compounds **1-5.** Transmission coefficient curves for **1-4** were reported in Ref 2). The HOMO resonance is predicted to be pinned near the Fermi Level of the electrodes for molecules **1** and **2** whereas, the LUMO resonance is predicted to be pinned near the Fermi level of the electrodes for **3-5**. In practice however, we expect Fermi Level to be in the vicinity of the mid gap for each molecule (black-dashed line), as shown in Figures S22-S31.

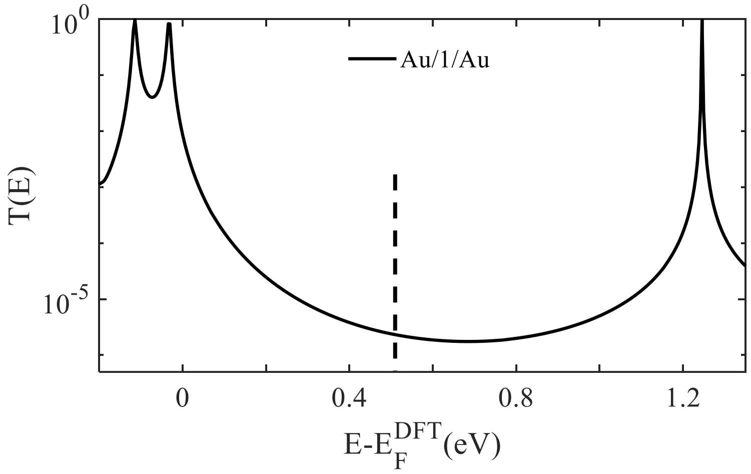

FIG S22. **Transmission coefficients of 1, (1,5-Di(4-(ethynylphenyl)thioacetate) anthracene.** Zero bias transmission coefficient T(E) **1,** (1,5-Di(4-(ethynylphenyl)thioacetate) anthracene, against electron energy *E*.

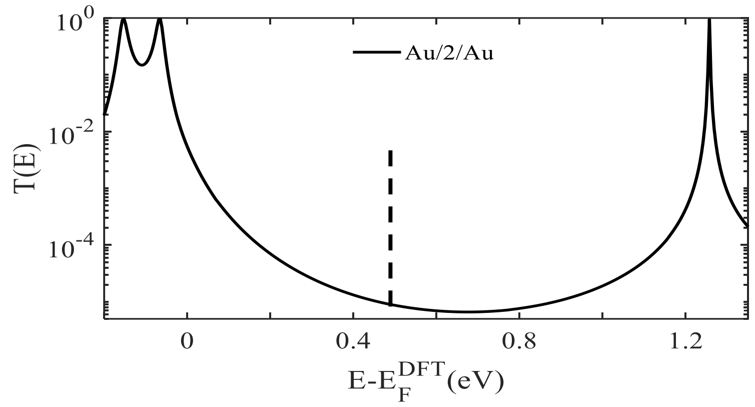

FIG S23. **Transmission coefficients of 2**, **(9,10-Di(4-(ethynylphenyl)thioacetate) anthracene.** Zero bias transmission coefficient T(E) of **2**, (9,10-Di(4-(ethynylphenyl)thioacetate) anthracene, against electron energy *E*.

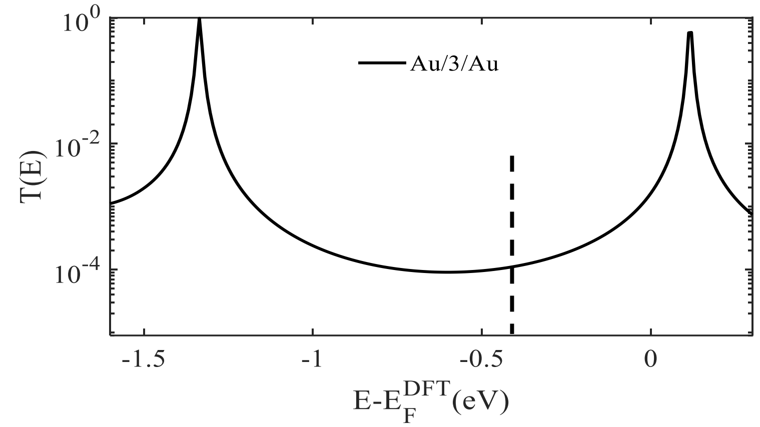

FIG S24. **Transmission coefficients of 3**, **(9,10-Di(4-ethynylthioanisole)anthracene**. Zero bias transmission coefficient T(E) of **3**, (9,10-Di(4-ethynylthioanisole)anthracene, against electron energy *E*.

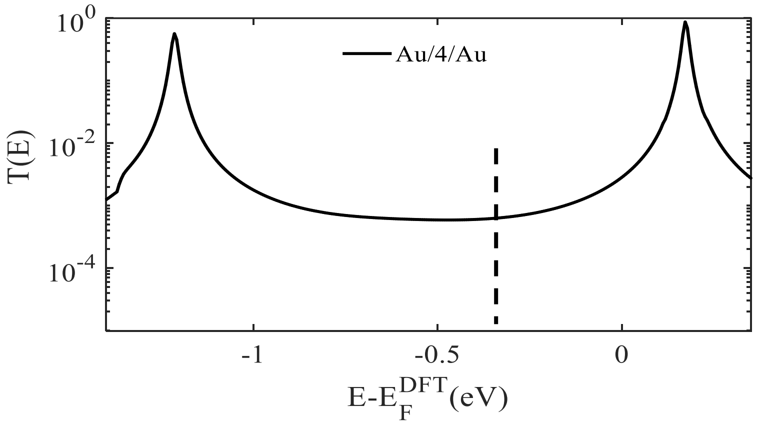

FIG S25. **Transmission coefficients of 4**, **(9-(4-(ethynylphenyl)thioacetate)-10-(4-ethynylpyridine) anthracene.** Zero bias transmission coefficient T(E) of **4**, (9-(4-(ethynylphenyl)thioacetate)-10-(4-ethynylpyridine)anthracene, against electron energy *E*.

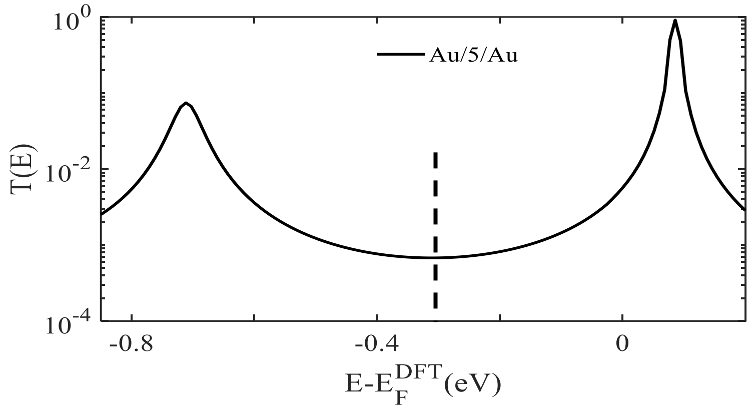

FIG S26. **Transmission coefficients of 5, (9-(4-ethynlthioanisole)-10-(4-ethynylpyridine)anthracene.** Zero bias transmission coefficient T(E) of **5**, (9-(4-ethynlthioanisole)-10-(4-ethynylpyridine)anthracene, against electron energy *E*.

3.6 Seebeck coefficients

To calculate the Seebeck cofficient of these molecular junctions, it is useful to introduce the non-normalised probability distribution $P(E)$ defined by

$$P(E) = -T(E)\frac{df(E)}{dE} \quad \text{(S2)}$$

where $f(E)$ is the Fermi-Dirac function and $T(E)$ are the transmission coefficients and whose moments L_i are denoted as follows

$$L_i = \int dEP(E)(E - E_F)^i \quad \text{(S3)}$$

where E_F is the Fermi energy. The Seebeck cofficient S, is then given by

$$S(T) = -\frac{1}{|e|T}\frac{L_1}{L_0} \quad \text{(S4)}$$

where e is the electronic charge and T is the temperature.

Supplementary Figures S27 and S31 show the Seebeck coefficient S evaluated at room temperature for different values of E_F, relative to the DFT-predicted Fermi energy E_F^{DFT}.

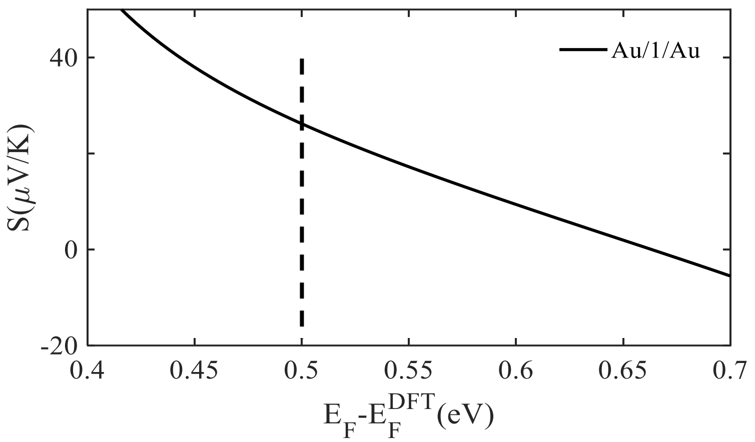

FIG S27. Seebeck coefficient S as a function of Fermi energy E_F for **1,** (1,5-Di(4-(ethynylphenyl)thioacetate) anthracene.

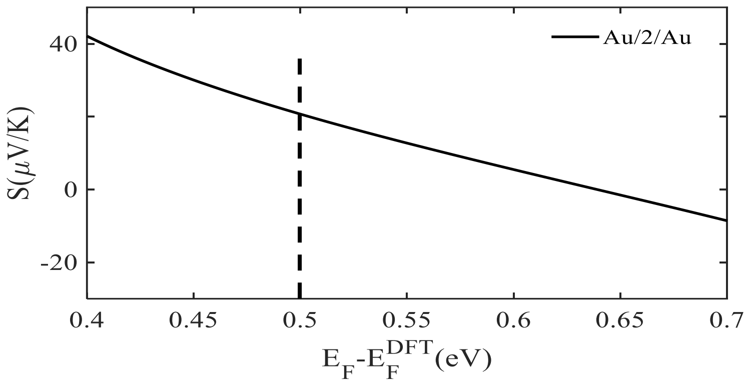

FIG S28. Seebeck coefficient S as a function of Fermi energy E_F for **2**, (9,10-Di(4-(ethynylphenyl)thioacetate) anthracene.

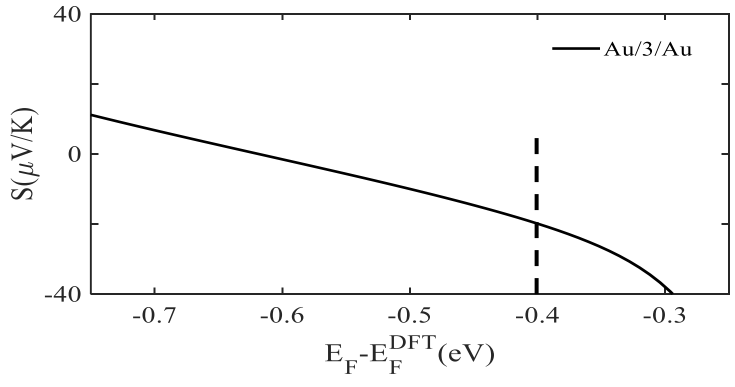

FIG S29. Seebeck coefficient S as a function of Fermi energy E_F for **3**, (9,10-Di(4-ethynylthioanisole) anthracene.

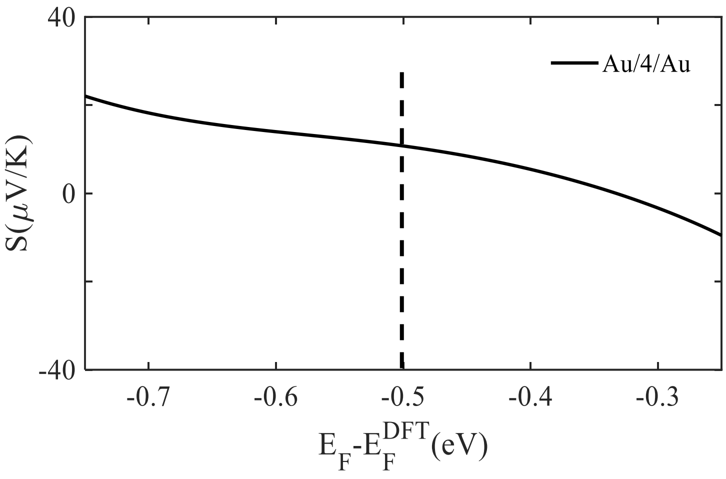

FIG S30. Seebeck coefficient S as a function of Fermi energy E_F for **4**, (9-(4-(ethynylphenyl)thioacetate)-10-(4-ethynylpyridine)anthracene**.**

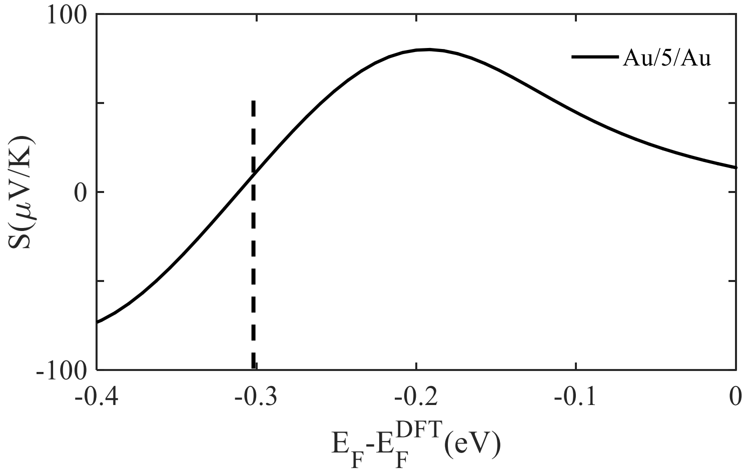

FIG S31. Seebeck coefficient S as a function of Fermi energy E_F for **5**, (9-(4-ethynlthioanisole)-10-(4-ethynylpyridine) anthracene**.**

3.7 Comparison between transport properties obtained using Au-Au and Pt-Au electrodes.

Figs. S32, S34 and S36 (molecules 1, 2 and 4), show that the both junctions yield approximately the same HOMO-LUMO transmission curves (red and black). However, red curves (Pt-Au junction) are downshifted towards a lower energy by about 0.2 eV, reflecting their different electron affinities of Au=223 and Pt=205 kJ/mole. The 0.2 eV shift causes the slope of the two curves (red and black) of molecule 4, to switch from positive (black) to negative (red), as shown in the yellow-dotted rectangle box. Since the slopE of the two curves change then the Seebeck coefficient must switch sign and that is clearly shown in Fig. S37.

The case for molecules **1** and **2** is slightly different, where the 0.2 eV shift causes the slopes of the two curves (red and black), to remain unchanged or to become flat (zero, red), as shown in the yellow-dotted rectangle box. Since the slopes of the two curves are unchanged their the Seebeck coefficients remain positive or approach zero, as shown in Figs. S33 and S35 (molecules **1** and **2**). This behaviour reflects the nature of the anchor group and whether the molecule is symmetric or asymmetric.

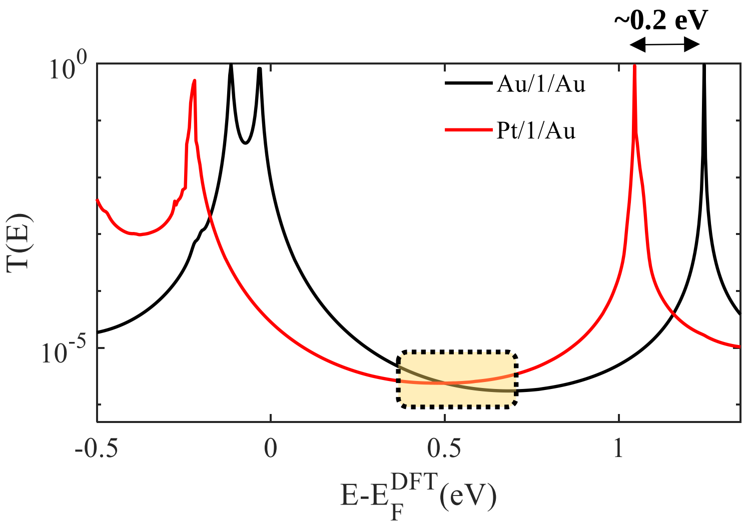

FIG S32. Zero bias transmission coefficients of **1** against electron energy *E* in two metallic junctions Au-Au (black lines), and Pt-Au (red lines).

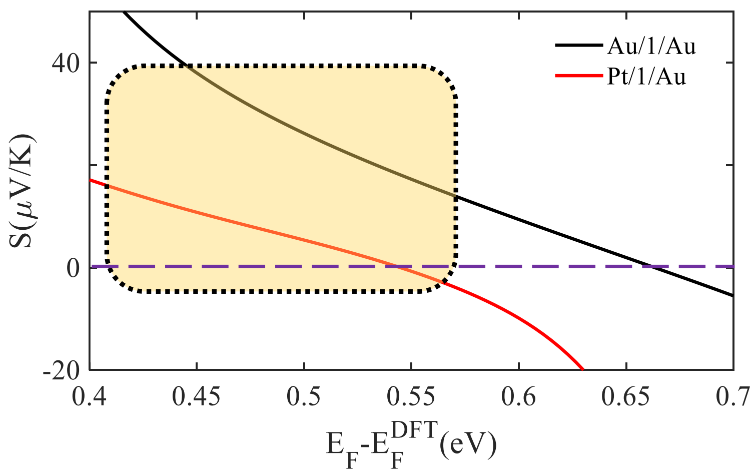

FIG S33. Seebeck coefficients S as a function of Fermi energy E_F for anthracene **1** in two junctions Pt/**1**/Au and Au/**1**/Au. Mainly positive S of Au-Au junction (black curve), mainly positive S of Pt-Au junction (red curve).

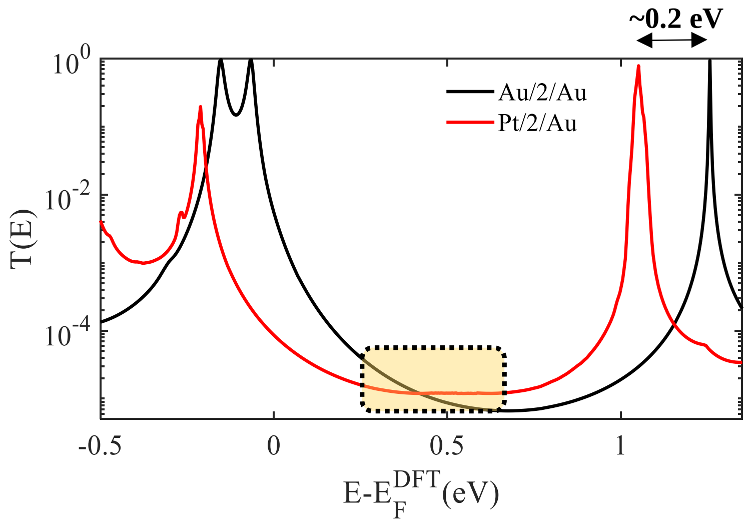

FIG S34. Zero bias transmission coefficients of **2** in two different junctions against electron energy *E* in two metallic junctions Au-Au (black lines), and Pt-Au (red lines).

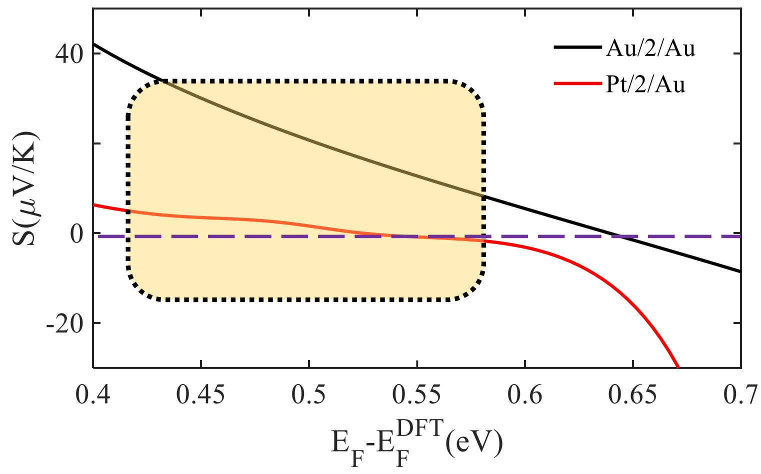

FIG S35. Seebeck coefficients S as a function of Fermi energy E_F for anthracene **2** in two junctions Pt/**2**/Au and Au/**2**/Au. Mainly positive S of Au-Au junction (black curve), mainly positive S of Pt-Au junction (red curve).

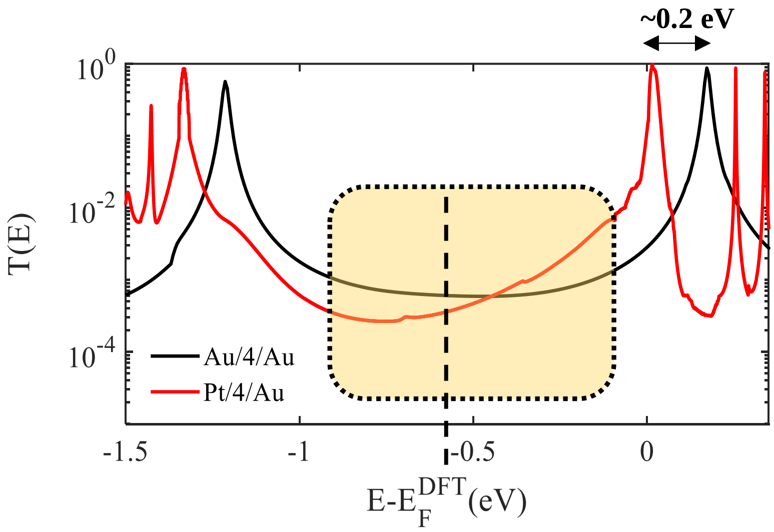

FIG S36. Zero bias transmission coefficients of **4** against electron energy *E* in two metallic junctions Au-Au (black lines), and Pt-Au (red lines).

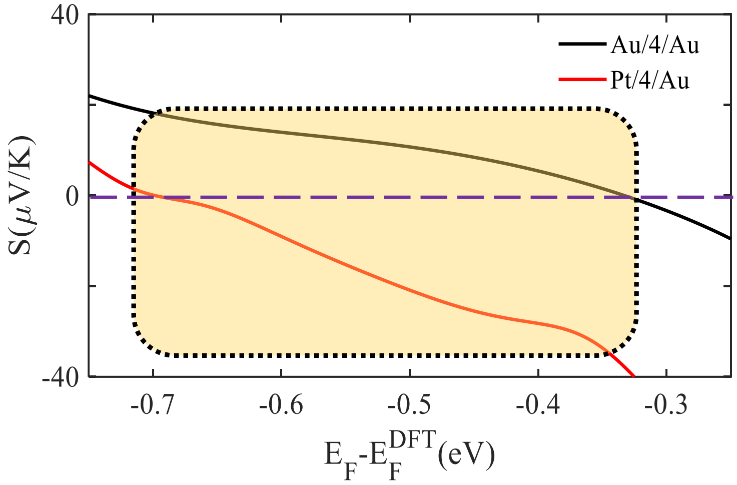

FIG S37. Seebeck coefficients S as a function of Fermi energy E_F for anthracene **4** in two junctions Pt/**4**/Au and Au/**4**/Au. Mainly positive S of Au-Au junction (black curve), mainly negative S of Pt-Au junction (red curve).

[1] C. Wang, A. S. Batsanov and M. R. Bryce, *J. Org. Chem.*, 2006, **71**, 108–116.

[2] X. Wang, T. L. R. Bennett, A. Ismael, L. A. Wilkinson, J. Hamill, A. J. P. White, I. M. Grace, O. V. Kolosov, T. Albrecht, B. J. Robinson, N. J. Long, L. F. Cohen and C. J. Lambert, *J. Am. Chem. Soc.*, 2020, **142**, 8555-8560.

[3] A. Ismael, X. Wang, T. L. R. Bennett, L. A. Wilkinson, B. J. Robinson, N. J. Long, L. F. Cohen and C. J. Lambert, *Chem. Sci.*, 2020, **11**, 6836-6841.

[4] L. E. Wilson, C. Hassenrück, R. F. Winter, A. J. P. White, T. Albrecht and N. J. Long, *Eur. J. Inorg. Chem.*, 2017, **2017**, 496–504.

[5] Soler, J. M.; Artacho, E.; Gale, J. D.; García, A.; Junquera, J.; Ordejón, P.; Sánchez-Portal, P., The SIESTA method for ab initio order- N materials simulation. *J. Phys.: Condens. Matter* **2002,** *14* (11), 2745.

[6] Artacho, E.; Anglada, E.; Diéguez, O.; Gale, J. D.; García, A.; Junquera, J.; Martin, R. M.; Ordejón, P.; Pruneda, J. M.; Sánchez-Portal, D. J. J. o. P. C. M., The SIESTA method; developments and applicability. **2008,** *20* (6), 064208.

[7] Ferrer, J.; Lambert, C. J.; García-Suárez, V. M.; Manrique, D. Z.; Visontai, D.; Oroszlany, L.; Rodríguez-Ferradás, R.; Grace, I.; Bailey, S.; Gillemot, K., GOLLUM: a next-generation simulation tool for electron, thermal and spin transport. *New Journal of Physics* **2014,** *16* (9), 093029.

[8] Milan, David C., Maximilian Krempe, Ali K. Ismael, Levon D. Movsisyan, Michael Franz, Iain Grace, Richard J. Brooke et al. "The single-molecule electrical conductance of a rotaxane-hexayne supramolecular assembly." *Nanoscale* 9, no. 1 (2017): 355-361.

[9] Ismael, Ali K., and Colin J. Lambert. "Single-molecule conductance oscillations in alkane rings." *Journal of Materials Chemistry C* 7, no. 22 (2019): 6578-6581.

[10] Naghibi, Saman, Ali K. Ismael, Andrea Vezzoli, Mohsin K. Al-Khaykanee, Xijia Zheng, Iain M. Grace, Donald Bethell, Simon J. Higgins, Colin J. Lambert, and Richard J. Nichols. "Synthetic control of quantum interference by regulating charge on a single atom in heteroaromatic molecular junctions." *The journal of physical chemistry letters* 10, no. 20 (2019): 6419-6424.
